# Luminescent Carbon Dots from Wet Olive Pomace: Structural Insights, Photophysical Properties and Cytotoxicity

**DOI:** 10.3390/molecules27196768

**Published:** 2022-10-10

**Authors:** Diogo A. Sousa, Luís F. V. Ferreira, Alexander A. Fedorov, Ana M. B. do Rego, Ana M. Ferraria, Adriana B. Cruz, Mário N. Berberan-Santos, José V. Prata

**Affiliations:** 1 Department of Chemical Engineering, Instituto Superior de Engenharia de Lisboa, Instituto Politécnico de Lisboa, 1959-007 Lisbon, Portugal; 2 CQ-VR-Centro de Química-Vila Real, Universidade de Trás-os-Montes e Alto Douro, 5001-801 Vila Real, Portugal; 3 BSIRG-iBB-Institute for Bioengineering and Biosciences, and Associate Laboratory i4HB—Institute for Health and Bioeconomy, Instituto Superior Técnico, Universidade de Lisboa, 1049-001 Lisbon, Portugal

**Keywords:** olive mill waste, hydrothermal carbonization, carbon dots, fluorescence, two-photon absorption, cytotoxicity, antioxidant activity

## Abstract

Carbon nanomaterials endowed with significant luminescence have been synthesized for the first time from an abundant, highly localized waste, the wet pomace (WP), a semi-solid by-product of industrial olive oil production. Synthetic efforts were undertaken to outshine the photoluminescence (PL) of carbon nanoparticles through a systematic search of the best reaction conditions to convert the waste biomass, mainly consisting in holocellulose, lignin and proteins, into carbon dots (CDs) by hydrothermal carbonization processes. Blue-emitting CDs with high fluorescence quantum yields were obtained. Using a comprehensive set of spectroscopic tools (FTIR, Raman, XPS, and ^1^H/^13^C NMR) in combination with steady-state and time-resolved fluorescence spectroscopy, a rational depiction of WP-CDs structures and their PL properties was reached. WP-CDs show the up-conversion of PL capabilities and negligible cytotoxicity against two mammalian cell lines (L929 and HeLa). Both properties are excellent indicators for their prospective application in biological imaging, biosensing, and dynamic therapies driven by light.

## 1. Introduction

Wet pomace (WP) is a semi-solid waste produced in large amounts during the two-phase extraction method of olive oil, which is currently viewed as the most eco-friendly extraction procedure, due to the drastic reduction of the process water compared to former methodologies [1]. By this method, around 800 kg of WP is produced per tonne of processed olives [2]. To put these figures into perspective, it suffices to say that over 3 million tonnes of olive oil are produced annually in the world economy [3]; therefore, the amount of olive mill by-products (pomace and wet pomace) easily reaches the astonishing amount of approximately 12 million tonnes per year. In many production countries, such as those of European Union (EU), the wet pomace is taken to specialized industrial units where the remaining oil is further solvent-extracted, yielding a raffinate oil, later used as an edible oil, and a solid residue that is used as fuel for energy production. WP is rich in a variety of natural products which include (poly)saccharides, lignin, (poly)phenols, lipids, and proteins as their main constituents [2]. Hence, it was anticipated by us that this residue would be very well suited to be realistically used as a renewable and sustainable resource for the synthesis of carbon-based nanomaterials, meanwhile reducing the environmental impacts of olive oil production [4,5,6]. The current work was focused on the synthesis of luminescent carbon nanoparticles having lateral sizes < 10 nm, commonly known as carbon dots (CDs), owing to their apparent shapes under TEM observation. A great number of reviews exist on the methods of synthesis and applications of CDs [7,8], including those pertaining to biomass-based CDs [9], and thus the overall subject will not be treated here. The great interest on CDs comes in the first place from their outstanding luminescence properties and photostability, which is a feature that is shared by semi-conductor quantum dots (SC-QDs), although the origin of fluorescence in both materials is not necessarily the same. Indeed, while in SC-QDs quantum confinement effects regulates the observed emission [10], the photoluminescence of CDs is still a subject that continues to warrant further investigation, despite the progress already achieved [11,12,13,14,15]. Beyond that, a major difference separates SC-QDs from CDs. Indeed, while CDs are usually considered biocompatible materials, and with generally low levels of cytotoxicity, the same do not apply to SC-QDs, which strongly hampers their use in biological fields [16], bolstering by this way the applications of CDs not only in nanomedicine but in a wide variety of related fields [9].

The synthesis of CDs from several biomass wastes has been accomplished in the last few years [17,18,19,20]. Examples include the use of wastepaper [21], wheat straw [22], food waste [23], tobacco leaves [24], rice residues [25], and wastewaters from olive mills [26] and the cork industry [27]. Besides our own investigations [26], research focused on olive mill wastes for preparing CDs is scarce but has been pursued by other groups. Luque et al. [28] have used olive pits under an acid-catalyzed (trifluoroacetic acid and sulphated zirconia) hydrothermal method (200 °C), while a pyrolysis treatment (600 °C) of olive solid wastes followed by wet oxidation (H_2_O_2_) was used by Valli et al. [29]. In yet another approach, Brachi [30] treated olive pomace hydrothermally (200–300 °C) in the presence of g-alumina. A blue emission is a general characteristic of these CDs, in instances dependent on the nature of the acid loadings [28]. For the CDs prepared by pyrolysis/oxidation [29], a quantum yield (QY) of 0.03 was obtained, while for the other two reports [28,30], no QYs were supplied; the mean sizes of CDs obtained by these methods were 4.4 nm [28], 2.8 nm [29], and 2–4 nm [30], as evaluated by TEM analysis.

In the study here presented, the synthetic efforts were directed towards excelling the PL of CDs while simultaneously departing from materials whose fluorescence has a strong contribution from molecular fluorophores [15]. For that purpose, and on a demonstration basis, we have deliberately extended the time and temperature of the hydrothermal carbonization (HTC) process. As will be shown, WP-CDs synthesized at high temperatures (250 °C) and prolonged reaction times (>16 h) do have high fluorescence QY (~0.2). Comprehensive spectroscopy studies were undertaken to unveil the structural features of the synthesized nanoparticles and establish their main photophysical properties. Studies concerning in vitro cytotoxicity have revealed that WP-CDs are highly biocompatible. Their antioxidant activity was also surveyed.

## 2. Results and Discussion

### 2.1. Characterization of Wet Pomace

**Scheme 1:**
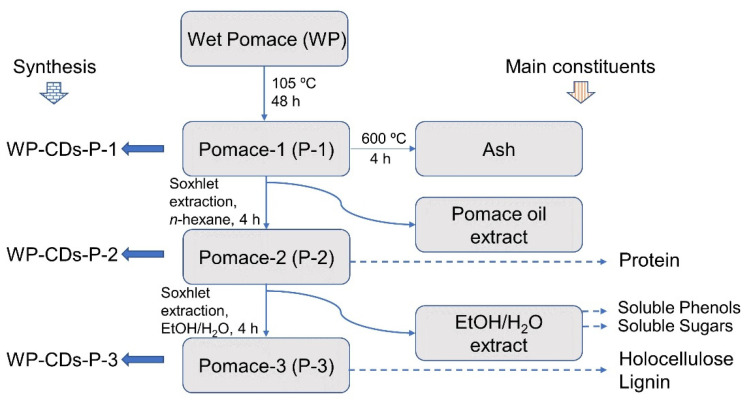
Fractioning of wet pomace and its main constituents.

The wet pomace used for WP-CDs synthesis was characterized for its physico-chemical properties (Table 1) and macro-organic constituents (Table 2), according to Scheme 1 (detailed experimental procedures may be found in the Materials and Methods section). The as-collected WP has a moisture content of around 70% and an acidic pH (5.5, from total dissolved solids (TDS)). On a dry basis, more than 80% of the residue (total suspended solids (TSS)) is not soluble in water, and its ash content is around 5%. Holocellulose (~40%) is the main organic constituent of WP, followed by lignin (~33%), while the triglycerides and proteins represent approximately 13% and 5%, respectively.

A significant amount of alcoholic/aqueous extractives (~14%), comprising soluble sugars and phenols, was also found. The organic composition found for WP is in accordance with reported values [2].

### 2.2. Synthesis of WP-CDs

As stated before, efforts were undertaken to select the best conditions to produce CDs whose photoluminescence resulted from the interplay of the carbon core states (herein meaning arene/heteroarene domains) with that of O/N containing substituents populating the CDs’ inner core or the surface, hence excluding as much as possible the emission being derived from molecular fluorophores. With that in mind, a comprehensive set of reaction conditions, favoring high HTC temperatures (≥250 °C) and extended times of reaction (≥4 h) were investigated. The suitability of P-1, P-2, and P-3 as carbon sources for the synthesis of WP-CDs was first addressed, given their different compositions (see above). Additionally, an extracted pomace sample (P-Ind) retrieved from an industrial unit was also used for comparison, since its composition should parallel that of P-2. The sift experiments were guided by HTC conditions formerly employed on the synthesis of CDs from olive mill wastewaters [26]. Table 3 gathers the results obtained, highlighting the CDs and hydrochar (HChar) mass yields, and QYs of as-synthesized CDs (cf. Materials and Methods section). The UV-Vis and fluorescence emission spectra of WP-CDs prepared from P-1, P-2, P-3 and P-Ind are depicted in Figure S1 (see Supplementary Materials).

On looking at the results on Table 3, one may notice that, in the first place, all pomace sources gave similar results (chemical yields of CDs and HChar, and CDs’ QYs) when the reaction temperature was fixed at 250 °C (highlighted in the Table). Secondly, lower reaction temperatures (200 °C) do not favor the enhancement of QY. Under this condition, a higher yield of HChar is however obtained, since part of the biomass components, namely cellulose and lignin, showed limited degradation at this temperature [31]. On increasing the temperature to 250 °C and beyond, the yield of gaseous products (mainly CO_2_, with CO, H_2_ and CH_4_ as minor components) rapidly builds up [31,32]. Since P-2 mimics the composition of the industrial pomace (after drying and solvent extraction, P-Ind), this was perceived as being the most interesting pomace fraction to be converted into CDs. Following this approach, an extended valorization of wet pomace may be achieved, which, besides furnishing on an industrial scale the already commercialized raffinate oil, would allow for a step further in the rational use of the extracted pomace, possibly contributing to the reduction of the environmental impacts of the olive oil industry. Needless to say, this residue (P-Ind) is highly virtuous because it is localized, accessible, and abundant. Alternatively, P-3 also constitutes a possible advantageous resource target, as far as practical applications of the alcoholic/aqueous extract are found (e.g., as antioxidants; see below). For the reasons outlined above, P-2 was selected as the representative pomace substrate for CDs synthesis under a large set of reaction conditions. These will be discussed along with the CDs characterization studies to assess their impact on the structure and PL properties of CDs.

### 2.3. Structural and Morphologic Characterization of WP-CDs

The structural analysis that follows was carried out on CDs purified by liquid-liquid extraction (denoted as-purified CDs; cf. Materials and Methods section for details), except where noted otherwise.

#### 2.3.1. FTIR

**Figure 1:**
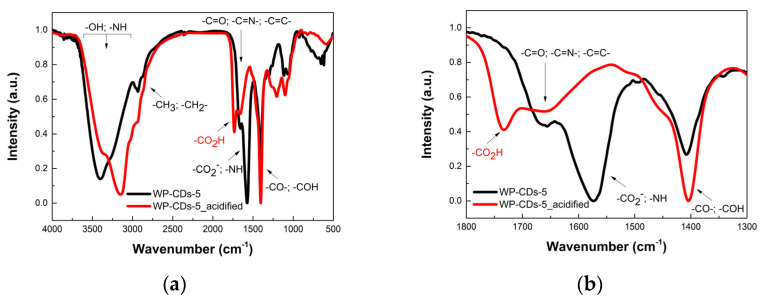
(**a**) FTIR spectrum of WP-CDs-5 (black line) synthesized using a [P-2] = 0.16 g/mL, an EDA/P-2 mass ratio = 0.08, at 250 °C during 72 h, overlayed with that of WP-CDs-5 obtained after acidification (red line; see text). (**b**) The same spectra amplified in the region between 1800–1300 cm^−1^.

Surface functionalities of WP-CDs were analyzed by FTIR spectroscopy using nanoparticles’ samples prepared under a diversified set of reaction parameters (dwell time, temperature, EDA/P mass ratio, and concentration of P). In the following, the WP-CDs-5 sample (72 h of reaction; Figure 1 and Table S1) was taken as the reference material for band assignments [33].

The O-H stretching (str) vibrations appeared as a very strong (vs) broad band centered at 3400 cm^−1^, along with a N-H str shoulder at 3260 cm^−1^, both H-bonded. Vibrations assignable to aliphatic/aromatic methyl and methylene groups appeared at 2965 cm^−1^ (CH_3_, str asymmetric (asym), very weak (vw); shoulder (sh)), 2934 cm^−1^ (CH_2_, str asym, w), 2880 cm^−1^ (CH_3_, str sym, vw; sh), 2858 cm^−1^ (CH_2_, str sym, vw; sh), while the corresponding bending (ben) vibrations appear at 1460 cm^−1^ (ben, vw). No aromatic C-H str bands were discernible. Three bands of strong to very strong intensity dominate the spectra of WP-CDs in the region between 1670–1400 cm^−1^, each of them the result of several group contributions. The first, appearing centered at 1660 cm^−1^ (m), was interpreted as being derived from overlapped stretching vibrations of C=O in *N*-substituted amides/primary amides, carbamates or ureas, aromatic Schiff bases (C=N str), and pyridinic rings. In-plane skeletal vibrations of C=C of aromatic rings, as in graphene oxide (GO) and reduced GO [34], may also contribute to the absorption at the lower energy side of the band. The band centered at 1573 cm^−1^, the most intense, may originate from a strong asymmetric carboxylate stretching vibration, possibly accompanied with contributions from N-H (ben) in amides/carbamates/ureas, and pyridinic rings (str). The assignment of this band to CO_2_^−^ was made upon acidification of a WP-CDs-5 aqueous solution to pH 1.9 followed by boiling for 30 min, and acquisition of the corresponding spectrum upon taking the sample to dryness and drying (Figure 1). As may be observed, the band around 1570 cm^−1^ almost disappears, giving place to a new band peaking at 1735 cm^−1^ corresponding to the carboxylic acid function. This is accompanied by the intensification of the band at around 1408 cm^−1^, probably due to mixed contributions from C-O stretching and in-plane C-OH deformation (both absent in the original carboxylate). Additionally, the C-O str appears as a new band at 1204 cm^−1^, and a broad OH str around 3150 cm^−1^. On basification of the above acidified aqueous solution of WP-CDs-5 to pH 8.7, followed by sample drying, the original spectrum was restored (Figure S2). The combined deformation vibrations of CH_2_ linked to electron withdrawing groups (CO, CN) and olefins (C=CH_2_), O-H deformation in aliphatic/aryl-OH, and carboxylates (sym str), may be responsible for second most strong band appearing at 1408 cm^−1^. The weak band at 1117 cm^−1^ may be traced to the bending vibrations (C-H mixed with C=C) in aryl rings and C-O stretching in H-bonded phenols and tertiary aliphatic alcohols, and that at 1053 cm^−1^ to C-O stretching in other aliphatic alcohols, aryl ethers and carboxylates.

Figure S3 presents the spectra of WP-CDs obtained by varying the time of reaction from 4 h to 72 h. Measurement of the relative intensities of the bands at ~1580 cm^−1^ (υ_2_) and ~1660 cm^−1^ (υ_1_) have indicated that the Iυ_2_/Iυ_1_ ratio has a perceptible increase after 32 h of reaction (Table S1), suggesting an enhanced amount of carboxylate moieties in CDs. On increasing the temperature while fixing other parameters ([P-2] at 0.04 g/mL, the EDA/P-2 mass ratio at 0.08, and 4 h of reaction), the Iυ_2_/Iυ_1_ ratio changes from 0.89 at 200 °C to 1.17 at 250 °C, and 1.12 at 300 °C (Figure S4 and Table S2), showing again an enhanced proportion of carboxylation in the nanomaterials synthesized at or above 250 °C. The Iυ_2_/Iυ_1_ ratio changes from 1.35 to 0.84 on increasing the EDA/P-2 mass ratio from 0 to 0.30 (Figure S5 and Table S3). The progressive decrease of the frequency intensity ratio indicates that the band centered around 1655 cm^−1^ has a strong contribution from nitrogen functionalities (e.g., N-C=O and C=N; see above), which became the most prominent band above the EDA/P-2 mass ratio of 0.08, under the specific conditions used (250 °C, 4 h heating, [P-2] = 0.04 g/mL). It is noted that the best QY (0.145) in this series was reached at an EDA/P-2 mass ratio = 0.08. Specific infrared frequency assignments of CDs, usually dependent on the carbon sources and on the synthetic methods employed for their synthesis, may be found in the literature [7,8,9].

#### 2.3.2. Raman

The Raman spectra of disordered carbon materials have two characteristic vibrational modes: the G band having E_2g_ symmetry that involves the in-plane stretching of pairs of sp^2^ carbons, and the D band from the breathing mode of A_1g_ symmetry [35]. In graphite, the G mode appears around 1580 cm^−1^, whereas the D mode in disordered graphite peaks around 1355 cm^−1^, and it is related to the presence of fused (6-atom) aromatic rings [36]. The existence of a D band in carbon-based structures such as defected graphite/graphene, graphene oxide (GO), and functionalized graphene sheets (FGS), may be taken as a measure of the systems’ disorder or amorphization [35,36,37,38]. The synthesized WP-CDs display this type of bands in their Raman spectra (Figure S6), resembling in part those appearing in GO/FGS samples prepared from pristine graphite [38,39], or even carbon black [37], although the G bands in these materials are observed in the 1580–1600 cm^−1^ range, whilst in WP-CDs they appear at ~1560 cm^−1^. The D and G bands in WP-CDs are highly broadened and strongly overlapped, with FWHM of ~254 cm^−1^ and 140 cm^−1^ on average, respectively (Table S4), probably representing mixed contributions from several overlapping bands (see below an enhanced spectral analysis). By this approach (two-band fitting), it appears that prolonged reaction times (from 4 h to 72 h) do not have a strong effect on the position of G and D bands, which peak, on average, at 1557 cm^−1^ and 1348 cm^−1^, also showing similar FWHW along the series for each band (Table S4). Worthy of note is that the position of the G band at ~1560 cm^−1^ in all analyzed WP-CDs samples implies a significant presence of sp^3^ domains according to the model of amorphization of pure graphitic materials developed by Ferrari and Robertson [35]. Indeed, the position of the G band correlates extremely well with the ratio of intensities of D (I_D_) and G (I_G_) bands experimentally found (I_D_/I_G_ = ~1).

The above band assignments and interpretations relied on models developed for graphite-based materials, having besides that a low oxygen content [35]. It is expected that in WP-CDs other existing functionalities can modulate the position and intensities of the displayed bands. For example, in WP-CDs-5, the oxygen content exceeds 30 wt % (cf. XPS analysis).

**Figure 2:**
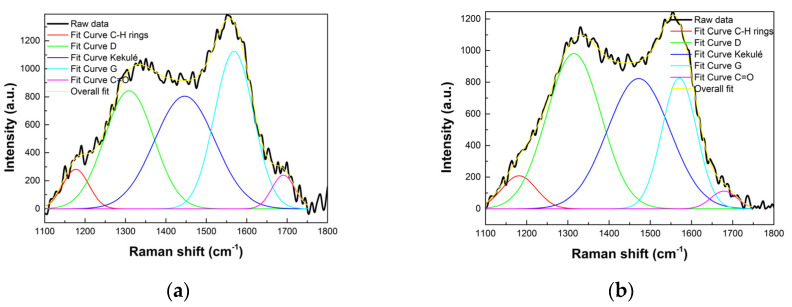
Decomposition of Raman spectra into five Gaussian components of (**a**) WP-CDs-1 and (**b**) WP-CDs-5, and the corresponding labelled bands.

The Raman spectra of WP-CDs were further interpreted following the proposals developed for glucose-based hydrochars (20–30 wt % oxygen) by Timko et al. [40]. The spectral analysis followed the five-band fitting approach proposed by Pöschl et al. [41] for the interpretation of Raman bands appearing on soot. Figure 2 presents the fitted band positions and Table 4 the FWHM and integrated band areas of each fitted band. The bands shown in Table 4 were named as CH-rings (C-H wagging on arene rings), D, Kekulé/furanic, G, and C=O (carbonyl). The D and G bands have the usual meanings, while the Kekulé band is originated in symmetric stretching vibrations of pairs of carbon atoms in arene rings, typically appearing in PAHs [40]. The correlations established in ref. [40] between the position and relative intensities of G and D bands with the number of aromatic rings within a structure indicate that the G and D bands appeared near 1570 cm^−1^ and 1310 cm^−1^ in a 16-ring arene, having an I_D_/I_G_ ~ 1. Moreover, the predicted Kekulé band for the same 16-ring system appeared at around 1440 cm^−1^; in the same spectral range, the C=C stretching of arene-furan fused rings (e.g., benzofuran and dibenzofuran) may also contribute (1440–1480 cm^−1^). The above data have a high degree of correspondence to what was found in WP-CDs (Table 4). An additional contribution to the band at ~1440 cm^−1^ may come from the symmetric stretching vibrations of carboxylate functionalities [42], for which a strong asymmetric stretching band was observed in FTIR. The areas of D and Kekulé bands are clearly increased on extended dwell times, supporting the higher degree of aromatization and carboxylation attained by the system. The foregoing analysis allows one to consider that WP-CDs are structures mainly composed by extended fused arenes/arene-furan rings interconnected to aliphatic (sp/sp^2^/sp^3^) carbon linkers/regions, which, on account of their area in visible Raman spectra (sum of G, Kekulé, and D band areas) represent most of the carbon in CDs.

#### 2.3.3. XPS

**Figure 3:**
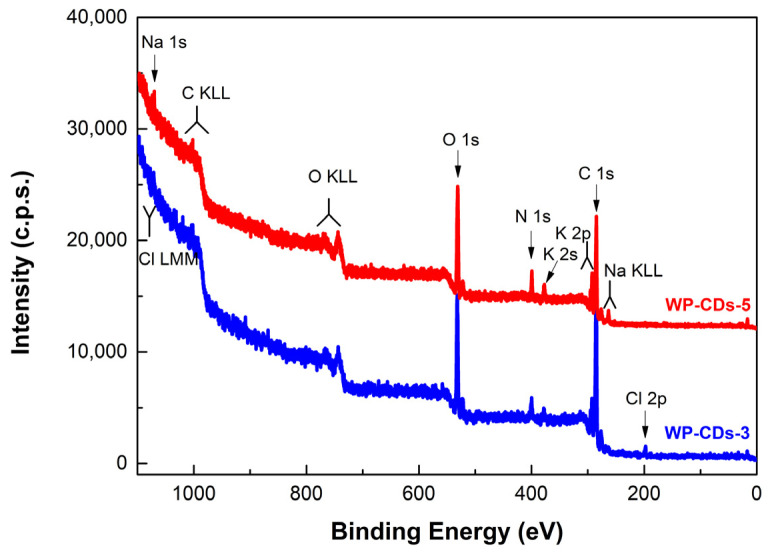
Wide XPS spectra of WP-CDs-3 and WP-CDs-5. Spectra intensities were offset for clarity’s sake.

**Figure 4:**
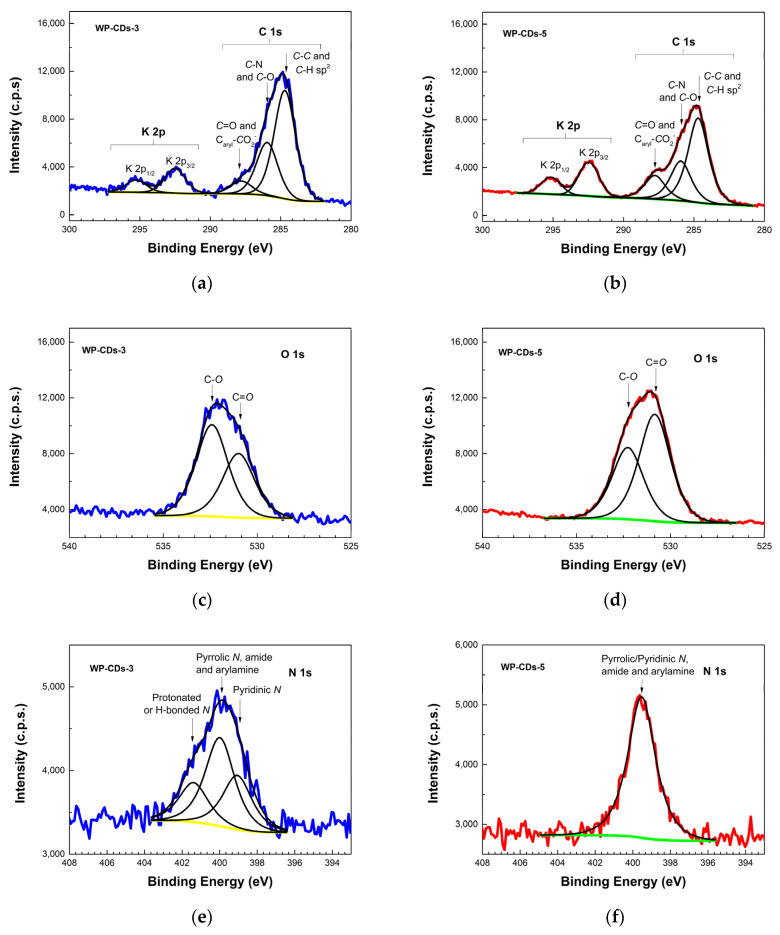
XPS spectra of WP-CDs-3 (**a**,**c**,**e)**; blue lines and WP-CDs-5 (**b**,**d**,**f)**; red lines showing C 1s and K 2p (**a**,**b**), O 1s (**c**,**d**), and N 1s (**e**,**f**) regions.

X-ray photoelectron spectroscopy (XPS) was used to survey the effect of the dwell time on the CDs’ elemental composition. Wide XPS spectra of WP-CDs-3 and WP-CDs-5 are shown in Figure 3. Carbon, oxygen, nitrogen, potassium, and chlorine are present in both samples. Assignments were made with reference to the equipment library [43], and reference databases [44,45]. The XPS characterization of each spectral region is given below (Figure 4). C 1s regions of both samples were fitted with three peaks centered at 284.7 ± 0.1 eV, 286.0 ± 0.1 eV, and 287.8 ± 0.1 eV. These peaks were assigned, respectively, to C-C and C-H sp^2^ carbon atoms (typical of arene rings), carbon bonded to nitrogen and singly bonded to oxygen (C-O), and carbon in carbonyl (C=O) and/or carboxylate groups bonded to aryl rings (C_aryl_–C(=O)O^−^) (Figure 4a,b)). The latter peak almost doubles in WP-CDs-5 (from 4.8 to 9.2 at. conc. %; cf. Table S5), showing a quite significant enhancement of carboxylation of the system for longer reaction times. O 1s regions also confirm the larger oxidation degree of WP-CDs-5 (Figure 4d): O 1s regions were fitted with two peaks centered at 530.9 ± 0.1 eV and at 532.4 ± 0.1 eV. The peak centered at lower BE is assigned to oxygen double bonded to carbon like in a carbonyl group, either bonded to aromatic carbon atoms (C_aryl_-C=O) or to nitrogen atoms (N-C=O), or oxygen double bonded to carbon in carboxylate groups (C_aryl_–C(=O)O^−^); the peak centered at higher BE is attributed to oxygen single-bonded to carbon in ether groups (C-O-C; aliphatic, arylic/furanic) and alcohols. It is noted that the relative intensities of these peaks clearly change for longer times of reaction: the peak identified as oxygen from carbonyl and carboxylate groups increases when compared to the one assigned to ether/alcohol groups (from 10.0 to 17.4 at. conc. %; cf. Table S5). Another difference between the 16 h and 72 h samples is revealed in N 1s regions (Figure 4e,f); while in WP-CDs-3 three different types of nitrogen groups were identifiable, namely pyridinic (399.1 ± 0.1 eV), pyrrolic, aryl amine and amide type groups (400.0 ± 0.1 eV), and protonated or H-bonded amines (401.4 ± 0.1 eV), a single peak centered at 399.5 ± 0.1 eV, possessing the same FWHM as those fitted for WP-CDs-3, was found in WP-CDs-5. This change may be attributed to a difference in the chemical environment around the nitrogen groups on prolonged heating periods, affecting mainly the pyridinic/pyrrolic/arylamine peaks.

The peak at 401.4 ± 0.1 eV is sometimes assigned to graphitic nitrogen. Such an assignment was not considered in our case because this peak disappears on prolonged heating (WP-CDs-5), which is contrary to what would be expected for such a stable moiety. Chlorine also changes for larger reaction times, being almost eliminated after 72 h of reaction (Table S6). A closer look into the detailed regions (Figure S7) shows that Cl 2p in WP-CDs-5, which is a simple doublet in WP-CDs-3 with a spin-orbit separation of 1.61 ± 0.1 eV assigned to Cl^−^, has an additional doublet, with the main component Cl 2p_3/2_ centered at 200.6 ± 0.1 eV. This was attributed to Cl bonded to carbon, indicating chlorination of the structures at longer times.

Potassium is clearly seen in both samples (Figure 4a,b). Although this potassium, with the K 2p_3/2_ component centered at 292.5 eV, may be bonded to Cl, the atomic ratio K/Cl > 1 (particularly in WP-CDs-5 where K/Cl >> 1), suggests that K^+^ is probably the counter-ion of a large number of carboxylate groups. Interestingly, also Na 1s, assigned to Na^+^, is detected in WP-CDs-5 (Figure 3), which also contributes to sample charge balance.

The overall results of XPS analysis have been summarized in Tables S5 and S6. The XPS peak assignments presented above are in qualitative agreement with the results found in Raman and FTIR analysis, such as the presence of extended sp^2^-hybridised domains in the synthesized CDs, along with a significant proportion of oxygen and nitrogen functionalities, particularly carboxylates. A reasonable correlation was also found between the quantitative analysis (wt %; Table S6) of WP-CDs-5 by XPS (which excludes hydrogen), and the elemental composition retrieved from microanalysis: C, 51.42%; H, 6.95%; N, 4.27%; S, 0.31%, and O, 37.05% (calculated), after correction for ashes (35%), to which corresponds an approximate empirical formula of C_41_H_64_N_3_O_23_.

#### 2.3.4. NMR

**Figure 5:**
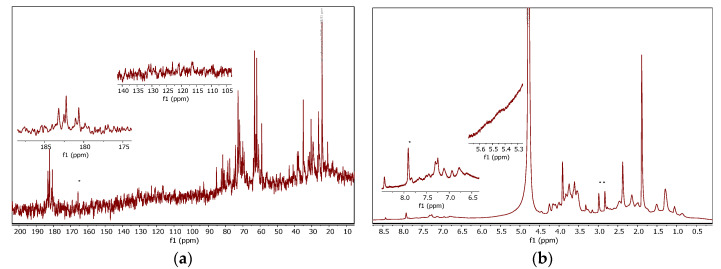
(**a**) ^13^C NMR and (**b**) ^1^H NMR spectra of WP-CDs-5 in D_2_O (32.5 mg/mL). Insets: Expanded regions of interest. Signals from DMF (5 mM), used as internal standard, are indicated by an asterisk (*).

^1^H/^13^C NMR spectra of WP-CDs-5 are shown in Figure 5. In the ^13^C NMR spectrum, at least three main regions are apparent. One, between ~20–40 ppm, shows resonances from aliphatic sp^3^-carbons, while that in the range 55–90 ppm comprise C-sp^3^ signals linked to heteroatoms (N/O) and, possibly, C-sp^2^/C-sp at the downfield side above 75 ppm. The third one is well denoted between 175–190 ppm, indicating the presence of several types of carboxyl derived carbon (N-C=O, O-C=O, CO_2_^−^) probably linked to aromatic domains existing in diverse environments within the carbon nanoparticles [46,47]. No signals were clearly discernible above 190 ppm, consistent with the absence of ketone resonances, in line with what was observed in FTIR analysis. The characteristic region of aryl carbons bonded to hydrogen or alkyl substituents is poorly represented in the spectrum. Weak signals attributable to aryl-fused furans and pyrroles or pyridinic derivatives may be observed in the region of 105–140 ppm, some of them being (~130 ppm) assignable to carbon resonances of fused aryl rings [48].

The ^1^H NMR spectrum corroborates the above assignments, displaying characteristic proton signals on saturated carbons (0.7–2.8 ppm), with those above ~1.8 ppm possibly linked to aromatic moieties and/or belonging to C-H resonances in β-carbons of saturated N/O heterocyclics. Protons on carbons linked to hetero atoms (N/O) appeared in the region 3.25–4.5 ppm. These last two regions account for almost 90% of quantifiable protons in the ^1^H NMR spectrum. Signals from olefinic C-H are barely seen (5.35–5.6 ppm). The low intensity proton resonances appearing between ~6.7–8.5 ppm may have their origin in aryl/aryl-fused 5/6-member ring heterocyclics (e.g., furan, pyrrole, and pyridine type).

#### 2.3.5. TEM and STEM

**Figure 6:**
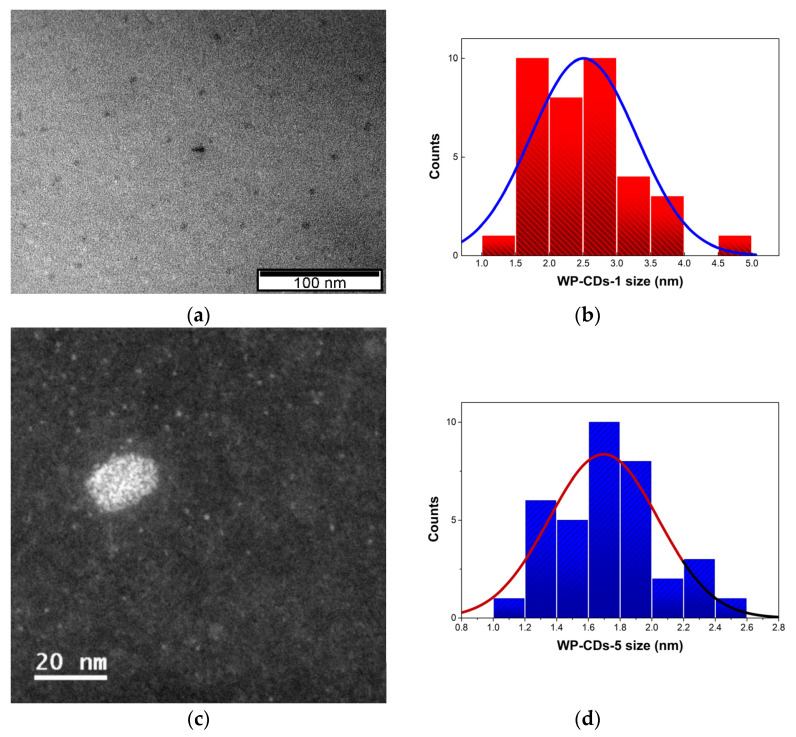
(**a**) TEM micrograph of WP-CDs-1 and (**b**) the corresponding histogram with the blue line representing the normal distribution. (**c**) STEM (HAADF detector) micrograph of WP-CDs-5 and (**d**) the corresponding histogram with the red line representing the normal distribution.

A TEM image of WP-CDs-1 is displayed in Figure 6a, showing quasi-spherical shaped particles with dimensions of 2.5 ± 0.8 nm. The reduced lateral dimensions of our nanoparticles allied to the inherent low contrast of carbon-based nanomaterials, or even their possible degradation under the high energy electron beams used during the TEM experiment [49], makes their observation difficult. Under our conditions of observation, part of the carbon nanoparticles in the sample may even be left unobservable. Indeed, carbon nanoparticles having molecular weights near or below 2.2 kDa, as determined by size-exclusion chromatography (SEC), were not seen with TEM [50]; this corresponds to particles of less than approximately 1.5 nm in diameter (calculation based on a graphite model [51]). In an attempt to improve the contrast and resolution, a STEM analysis was performed on the WP-CDs-5 sample. The results seem to be slightly better compared to TEM (cf. Figure 6c). A similar contour shape was observed for the nanoparticles, which have mean sizes of 1.7 ± 0.3 nm. The larger spot appearing in Figure 6c is likely the result of the aggregation of the nanoparticles during the drying process of the sample preparation. Further work is being carried out to correctly assess the sizes of WP-CDs by combining SEC methods with time-dependent anisotropy decay techniques.

### 2.4. Photophysical Properties of WP-CDs

#### 2.4.1. General 

**Figure 7:**
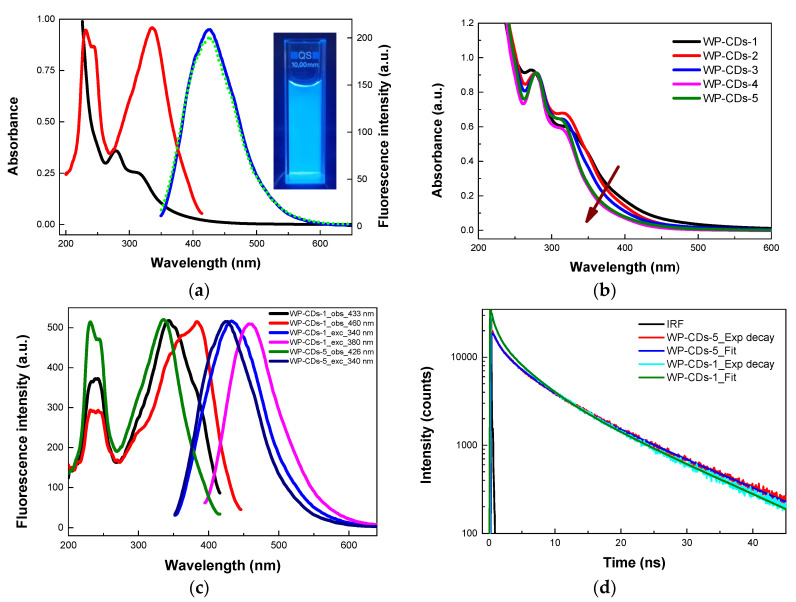
(**a**) UV–Vis (black line), excitation (red line; monitored at 426 nm) and emission (blue line, excitation at 340 nm; dot green line, excitation at 230 nm) spectra of an aqueous solution of WP-CDs-5 (0.1 mg/mL); inset: image of WP-CDs-5 under 365 nm illumination. (**b**) UV-Vis spectra of WP-CDs-1 to 5 (normalized at 278 nm). (**c**) Emission spectra of WP-CDs-1 excited at 340 and 380 nm and the corresponding excitation spectra monitored at the corresponding emission maxima; for comparison, the emission and excitation spectra of WP-CDs-5 are overlayed. (**d**) Time-resolved intensity decays (excitation at 340 nm; observation at 430 nm) of WP-CDs-1 and 5 obtained by single-photon timing (SPT) method.

The extension to which the hydrolysis of polymeric constituents (e.g., carbohydrates, lignin, proteins, and polyphenols) of pomace biomass occurs, and the condensation, polymerization, and carbonization reactions that follow during the HTC treatment, either in stepwise or simultaneous fashion, will dictate the UV-Vis profile and emissive properties of WP-CDs. The most characteristic bands in the UV–Vis spectrum of WP-CDs-5 (used as reference material) in aqueous solution (0.1 mg/mL) appear around 245 nm (as a shoulder), 278 nm, and 318 nm (Figure 7a), with an absorption cut-off near 600 nm.

The two bands peaking at higher energies may be assigned to π–π* transitions of sp^2^ hybridized carbon structures of conjugated olefin systems (3–4 units) and aromatic moieties. The band around 320 nm was attributed to extended π-conjugated systems (olefinic and aromatic), combined or not with certain functional groups (e.g., n–π* and π–π* mixed transitions of conjugated carbonyl, carboxyl, and imine functions). CDs prepared under reduced times of reaction (4 h, WP-CDs-1) displayed additional bands around 350 nm and 400 nm. These low energy absorptions are progressively reduced as the time of reaction was increased from 4 h to 72 h (WP-CDs-1-5; Figure 7b), the UV-Vis profile being essentially stabilized after 32 h. This may be an indication that, upon prolonged heating, the more labile structures react further, yielding more condensed carbon frameworks. When the CDs obtained at 200 °C, 250 °C and 300 °C under 4 h of reaction are compared, one may also perceive that the absorption in the region between 300–500 nm deeply decreases its intensity at the highest temperature (WP-CDs-6 to 8, Figure S8a), in line with what was observed at 250 °C but at a prolonged heating period. CDs prepared with increasing amounts of EDA (EDA/P-2 mass ratio between 0 and 0.30; WP-CDs-9 to 12) display a notorious absorption enhancement above 300 nm (Figure S8b), which is traced to an increase of chromophores possessing n–π* and π–π* mixed transitions.

Although the absorption range of WP-CDs may fall into the optical window typical of many CDs materials [7,8,9], showing the main absorption bands peaking between 250–450 nm, their shapes are, in instances, different from other reported CDs [29]. This is an obvious consequence of the different distribution of energy gaps attained by each nanomaterial prepared from diverse sources and methods, for which the core structure and the surface functionalities certainly play a role [7,8,9].

The excitation spectrum of WP-CDs-5 monitored at 426 nm shows the presence of a narrow band at 232 nm with a shoulder at 244 nm, and a broader band (FWHM = 0.91 eV) peaking at 337 nm, the latter being slightly intense (Figure 7a). A low intensity shoulder is also perceptible at ca. 380 nm. As may be seen from Figure 7a, the absorption and excitation spectra are not superimposable. This may stem in part from the formation of aromatic domains that, albeit possessing strong absorption below ~280 nm, are essentially non-emissive. The fluorophore largely responsible for the blue emission of WP-CDs has its absorption bands at ~240 nm and 320 nm. On excitation at 340 nm, the wavelength at which the emission is maximized, a strong emission band peaking at 426 nm is displayed by WP-CDs-5 (Figure 7a). The same emission spectrum is obtained upon excitation at 232 nm, showing that the excitation bands peaking at around these values (230–240 nm and 340 nm) belong to the same type of emitter (Figure 7a). A highly reproducible fluorescence quantum yield of 0.21 ± 0.02 was obtained for WP-CDs-5 from several batches (under excitation at 340 nm). Interestingly, the excitation spectrum of WP-CDs-1 exhibits, besides the main band peaking at ca. 340 nm, a shoulder at around 380 nm when the emission is monitored at ~430 nm, which becomes the excitation peak maximum on emission observed at 460 nm (Figure 7c). Indeed, for these CDs, a higher QY is registered at an excitation wavelength of 380 nm (QY = 0.19) as compared to that of 340 nm (QY = 0.15). A complete account of the QYs obtained for WP-CDs-1 to 12 may be found in Table S7. As with other optical properties, the wavelength of fluorescence emission maximum is highly dependent on the particular nature of the CDs nanoparticles (distribution and arrangement of core and surface functionalities) and may range from the violet to the red [9].

The time-resolved intensity profile of WP-CDs-5, obtained from the single-photon timing method (SPT), is shown in Figure 7d. The corresponding lifetimes were calculated by iteratively fitting the exponential functions to the multi-exponential model (see Materials and Methods section for details). It was found that the decays were best fitted to a sum of three exponentials (*τ*_1_ = 0.84 ns, *f*_1_ = 5.1%; *τ*_2_ = 4.5 ns, *f*_2_ = 30.2%; *τ*_3_ = 13.7 ns, *f*_3_ = 64.7%, where the *f*_i_ are the fractional contributions of each component), yielding an intensity average lifetime (*τ*_ave_) of 10.2 ns (χ^2^ = 1.30). It is worthy of note that the longest lifetime component in WP-CDs-1 (Figure 7d) has a lower contribution (*τ*_3_ = 12.9 ns, *f*_3_ = 54.7%) to the average lifetime, whilst the shortest lifetime component (*τ*_1_ = 0.87 ns, *f*_1_ = 9.3%) contributes more, leading to a *τ*_ave_ of 8.7 ns (χ^2^ = 1.39). Lifetimes of WP-CDs synthesized under other representative conditions have been collected in Table S8.

For all WP-CDs, the emission wavelength maxima shift towards the red on decreasing the excitation energy, always being accompanied by an intensity decrease (Figure S9, with WP-CDs-5 as an exemplar).

The pronounced reduction in PL intensity on increasing the excitation wavelength may be attributed to the decrease in absorption. The emission wavelengths varied linearly with the excitation wavelength, above 340 nm, with the corresponding fluorescence redshifts decreasing from 0.74 eV to 0.325 eV on going to lower energies (inset Figure S9a). Several models have been idealized to explain the emission tunability of CDs [14]. The one considering that each CD particle behaves as a single emitter has been supported by single-particle level experiments [12]. Accordingly, the dependence of WP-CDs emission on the excitation wavelength may be viewed as a result of the spectral heterogeneity of the single emitters, possessing different sizes and structural peculiarities, acting collectively. Furthermore, a strong dependence on the excitation wavelength was also found in the emission decays of WP-CDs. This is illustrated in Figure S9b, where the intensity decays of WP-CDs-5 upon excitation at 340 nm and 405 nm are shown. Under 405 nm excitation, a strong reduction on the contribution of the longest lifetime component (more than threefold) is observed (*τ*_3_ changes from 13.7 ns, with *f*_3_ = 64.7%, to 11.0 ns, with *f*_3_ = 19.6%), while the fastest components increase their contributions (*τ*_1_ = 1.5 ns, *f*_1_ = 16.1%; *τ*_2_ = 3.8 ns, *f*_2_ = 64.3%), yielding an average lifetime 4.8 ns (a decrease from *τ*_ave_ of 10.2 ns). These findings have a strong conceptual resemblance to those described by Si et al. for CDs derived from the laser ablation of graphite [52], for which different exciton-like radiative recombination routes were proposed to explain the fluorescence emission. Hence, excitation at the high energy side (e.g., at 230 nm and 340 nm) will directly excite both the carbon core (which has a larger energy gap) as well as part of the structural functionalities residing at the surface of the carbon core (which possess a lower energy gap). Following excitation, carbon core relaxation will lead to the slow decay observed, whereas the fast decay components will derive from the direct deexcitation of functionalities at surface sites. At longer wavelength excitations (405 nm in the example), the dominant contribution will come from the surface functionalities. The observed dependence of emission on excitation energy contrasts with the PL features of CDs highly dependent on molecular fluorophores (embedded or linked to the carbon matrix or its surface), for which the emission is not sensitive to excitation energy changes [15,53]. That the observed emission of WP-CDs at ~430 nm is primarily originated from surface functionalities (upon energy relaxation from core transitions onto surface functionalities, on excitation at 232 and 340 nm), and not directly from the aromatic domains residing more deeply in the condensed matrix, is supported by the following heavy atom quenching experiment, as others before us have used [11]. The steady-state Stern-Volmer (SV) plot (Figure S10) of the quenching of WP-CDs-5 emission upon addition of increasing amounts of NaI in thiosulphate solution, furnished a linear relationship (*K*_SV_ = 54.94 M^−1^; R^2^ = 0.9979) from which a bimolecular quenching constant of 5.4 x 10^9^ M^−1^ s^−1^ was retrieved, assuming an essentially dynamic mechanism. Such linearity is a strong indicator that most of the emission of WP-CDs comes from sites easily reachable by an external quenchers like iodide, pointing to their location at the particle surface.

The photostability of WP-CDs-5 (0.1 mg/mL) was evaluated at pH = 7.2 (phosphate buffered solution) under continuous irradiation (excitation at 340 nm) by a low power source (pulsed Xenon discharge lamp with an average power of 7.3 W) for ca. 2 h. Under these conditions, a remarkable photostability is secured (Figure S11).

#### 2.4.2. Two-Photon Absorption 

CDs featuring a two-photon absorption (TPA) mechanism may widen their application fields, such as in nanomedicine applications, through improved biological imaging and sensing, and deeper tissue penetration in photodynamic therapies [7]. This is fortunately the case with our WP-CDs which display this important property.

**Figure 8:**
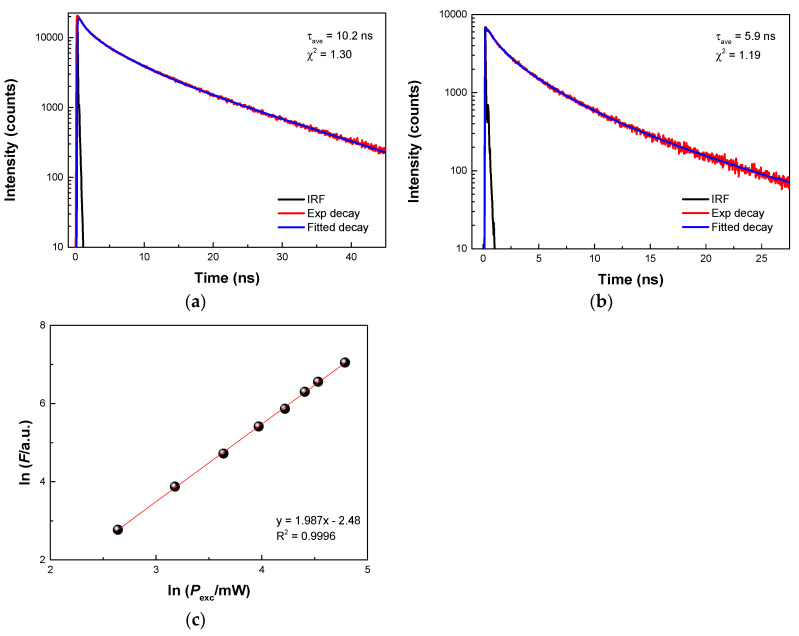
Time-resolved intensity decays of aqueous solutions of WP-CDs-5 under excitations at 340 nm (**a**) and at 620 nm (**b**), both emissions being observed at 430 nm; (**c**) demonstration of the quadratic dependence of emission characteristic of two-photon absorption by plotting the logarithm of fluorescence intensity (F) vs. the logarithm of the average excitation power (*P*_exc_). Excitation at 620 nm, emission at 430 nm.

For example, when excited by a laser source at a wavelength of 620 nm (red light), WP-CDs-5 nanoparticles emit blue light at 430 nm. The corresponding time-resolved decay profile is identical to that obtained under 340 nm excitation, monitored at 430 nm (Figure 8). Given that the excitation at 620 nm is monochromatic, this result clearly suggests the existence of an upconversion mechanism on these CDs. To confirm that this was indeed the case, the dependence of fluorescence intensity on the excitation intensity was measured. The average excitation power was between 14 mW and 120 mW. The pulsed laser used had a pulse width of 5 ps and a repetition rate of 3.4 MHz. Using an estimated beam diameter of 0.1 mm at the sample, the excitation peak intensity was estimated to be ca. 90 mW/cm^2^ for the maximum power used. The quadratic dependence observed for the fluorescence as a function of average excitation power (Figure 8c), together with the great redshifted emission with respect to excitation (ruling out triplet-triplet annihilation typical of molecular fluorophores; cf. steady-state fluorescence measurements above), unequivocally demonstrates that the WP-derived carbon nanomaterials have significant two-photon absorption. Determination of the TPA absorption spectra of WP-CDs is underway to further unravel their potential in the biomedical field.

#### 2.4.3. Emission Intensity vs. pH

An aqueous solution of WP-CDs-5 has a neutral pH (~6.8). On acidification to pH 1.9, the original band at 278 nm suffers a 2 nm redshift and increases its intensity, while the former band at 317 nm is decreased with the concomitant appearance of a band at around 350 nm, with a tailing up to 650 nm (Figure S12a). Ongoing to pH 8.7, the absorption maximum is kept around the values attained at pH ~ 7, but an overall decrease of absorbance is observed in this region. A new band is additionally formed at ~445 nm. An apparent isosbestic point is observed at 356 nm, reflecting the equilibrium between the several species on varying the pH. In all cases, the emission maximum is maintained at 426 nm (Figure S12b). A great PL stability is observed for WP-CDs-5 between pH 1 and ~8, a characteristic that is shared with the CDs obtained from the wastewaters of olive mills [26] and cork factories [27]. Upon increasing the pH above this value, the fluorescence drops abruptly, reaching only 45% of the pristine emission at pH 11.6 (Figure S12c PL intensity in most reported CDs is highly sensitive to pH changes. Such behavior is usually attributed to the ionization of functional groups (e.g., carboxylic acids, phenols, amines) present at the nanoparticle surface [7,8]; hence, a particular surface arrangement of basic/acid groups, which are in turn related to the raw materials and methods used in their synthesis, will modulate the observed PL stability. For this reason, some of the CDs show a linear PL dependence on the pH between ca. 2.0–8.0 [7], others keep the PL constant between 4.0–8.0 but decrease outside that range [8], and yet others see their PL intensity decrease below 4.0 but are stable up to pH ~11 [29].

#### 2.4.4. Emission Intensity vs. Concentration

**Figure 9:**
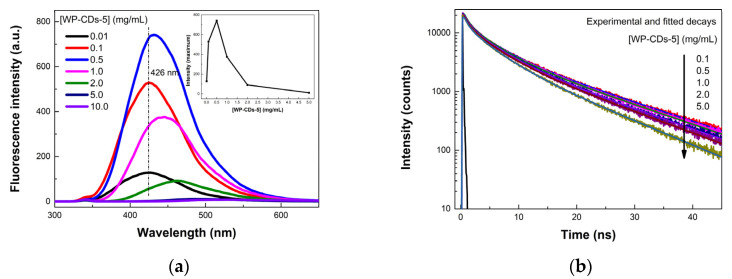
(**a**) Steady-state emission spectra and (**b**) time-resolved intensity decays of aqueous solutions of WP-CDs-5 at various concentrations, excited at 340 nm. Inset in (**a**): intensity (monitored at emission maximum) vs. concentration of CDs. Exponential decays observed near the emission maximum at each concentration (430, 435, 445, 460, and 500 nm, respectively).

The fluorescence QY of WP-CDs in aqueous solutions is deeply impacted by the concentration of nanoparticles. From 0.01 mg/mL up to 0.1 mg/mL, the PL intensity increases while the emission maximum is kept unaltered at 426 nm (Figure 9a). Going onward, the intensity still increases (at least up to 0.5 mg/mL) but a slight redshift (6 nm) on the emission maximum is observed. At higher concentrations (1–10 mg/mL), a pronounced decrease in fluorescence occurs, being accompanied by strong redshifts. For example, at 5 mg/mL, the remaining fluorescence represents only 1.5% of the highest recorded intensity (0.5 mg/mL), and its maximum is shifted by 60 nm. The PL practically vanishes above this concentration. The progressive bathochromic shifts were attributed to the increased aggregation of nanoparticles, which afford in this way successively lower energy gaps; concomitantly, the QY is also strongly reduced due to aggregation-caused quenching (ACQ) derived from π-π stacking of extended conjugated aryl moieties [54].

The decay profiles of WP-CD-5 at the various concentrations are shown in Figure 9b, under observation near the emission maxima at each concentration. The intensity average lifetimes progressively decrease from 10 ns (0.1 mg/mL) to 7.5 ns (5 mg/mL). Analysis of the three components’ decay has demonstrated that the longest one saw a reduction on its fractional contribution (from 64% to 57%), accompanied by a decrease in its lifetime (from around 14 ns to 10.6 ns), while the other two components proportionally increase their contributions but essentially kept their lifetimes. Lifetimes and fractional contributions for this set of experiments are gathered in Table S9.

### 2.5. Cytotoxicity of WP-CDs

**Figure 10:**
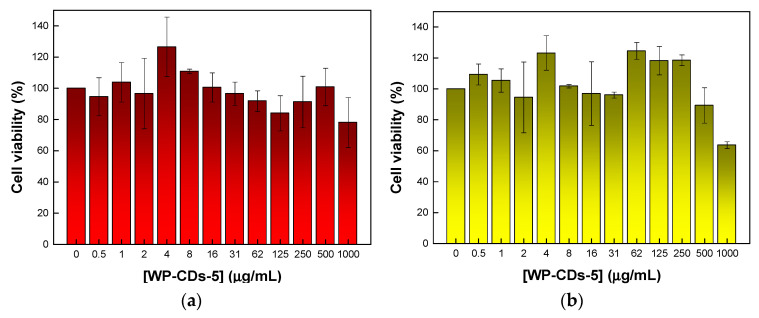
In vitro cytotoxicity results of WP-CDs-5 at various concentrations (0.5–1000 µg/mL) against (**a**) L929 and (**b**) HeLa cells after incubation for 24 h, as evaluated by the resazurin fluorescence viability assay.

The in vitro cytotoxicity of WP-CDs was evaluated by a resazurin fluorescence viability assay (PrestoBlue^TM^; details may be found in Materials and Methods section). The resazurin-based assay is a cell health indicator, giving information about the cell metabolic activity. Upon entrance into living cells, resazurin (a poorly fluorescent *N*-oxide phenoxazine dye) is reduced to resorufin, a highly fluorescent reduced phenoxazine. The transduction signal is proportional to mitochondrial enzymatic activity (metabolically active cells), which is quantitatively translated into cell viability. A non-tumoral mouse fibroblast cell line (L929) and a tumoral human cervical cancer cell line (HeLa) were used in the viability assays. As depicted in Figure 10, after being incubated with various concentrations of WP-CDs-5 (0.5–1000 µg/mL), both cell lines generally showed high viabilities after 24 h, up to a concentration of CDs of 500 μg/mL.

To discard the possibility of a direct chemical reduction of resazurin by WP-CDs, which would have implications in the collected cell viability values, a further experiment was conducted in the absence of cells. The results unequivocally showed that WP-CDs were not able to directly reduce the oxidized phenoxazine dye (Figure S13), since the fluorescence intensity of resazurin remains the same over the entire concentration range. Hence, the cell viability outcomes are not compromised. A decrease in cell viability (78% for L929 and 64% for HeLa cells) was only observed at a much higher concentration (1000 μg/mL), which is well over the one usually required for bioimaging applications. Furthermore, a cytotoxic effect should only be considered with a reduction of cell viability by more than 30% of the control (ISO EN 10993-5). These results support the non-toxic character of WP-CDs, demonstrating their excellent biocompatibility and therefore paving the way to their future use in biomedical fields.

### 2.6. Antioxidant Activity of WP-CDs

**Figure 11:**
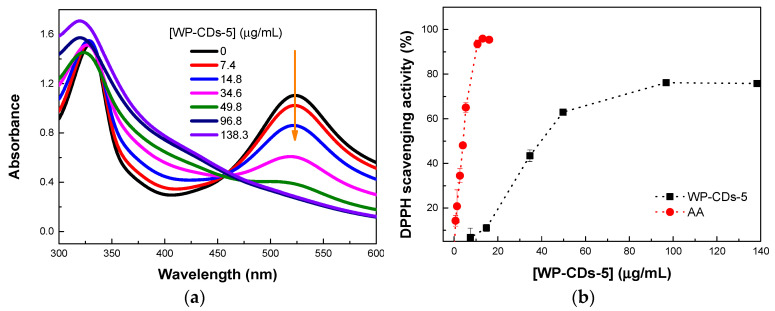
(**a**) Absorption spectra of DPPH in ethanol (0.127 mM) on increasing the concentration of WP-CDs-5 in the range 7.4–138 μg/mL and (**b**) the scavenging activity of DPPH radicals by WP-CDs-5, having AA for comparison (absorbance monitored at 520 nm). Dotted lines drawn as an eye guide.

The antioxidant properties of WP-CDs were evaluated against 2,2-diphenyl-1-picrylhydrazyl (DPPH) radical using a well-established colorimetric assay [55] (details may be found in the Materials and Methods section). Using WP-CDs-5 as a model CD in a concentration range of 7.4–138 μg/mL, the scavenging activity for DPPH radicals increased linearly with the concentration of CDs up to ~50 μg/mL (~65% of scavenging activity), saturating after that (Figure 11). From the linear region of the plot, a half-maximal inhibitory concentration (IC_50_) of 38.2 μg/mL was retrieved, which compares to a value of 4.2 μg/mL for ascorbic acid (AA), a potent antioxidant. The antioxidant activity shown by WP-CDs toward the DPPH radical is noteworthy, being the IC_50_ better or comparable to those reported in recent studies with CDs [56,57,58]. The scavenging radical activity assay performed on the alcoholic/aqueous extract from Pomace-2 (cf. Scheme 1) has also demonstrated a significant antioxidant activity, with an IC_50_ of 41.4 μg/mL, suggesting it as a possible source of antioxidants [59].

## 3. Materials and Methods

### 3.1. General

Wet pomace (WP) was collected from an industrial olive extraction unit (olive mill) at Ribatejo, Portugal, operating on a two-phase centrifugation process. The WP was kept refrigerated at −15 °C in polyethylene bags until use. An industrial sample of dried and extracted (*n*-hexane) wet pomace (P-Ind) was collected from a pomace oil recovery unit at Alentejo, Portugal.

Quinine hemisulphate monohydrate (> 98%, Fluka, Sigma-Aldrich Corp., St. Louis, MO, USA), ethylenediamine (EDA, >99.5%, Fluka, Sigma-Aldrich Corp., St. Louis, MO, USA), 2,2-diphenyl-1-picrylhydrazyl (DPPH, 95%, Alfa Aesar, Kandel, Germany), L-ascorbic acid (AA, 99%, VWR, Radnor, PA, USA), gallic acid (97.5%, Sigma, Sigma-Aldrich Corp., St. Louis, MO, USA), D-glucose monohydrate (for biochemistry and microbiology, Merck, Darmstadt, Germany), *N*,*N*-dimethylformamide (DMF, >99.8%, Sigma-Aldrich Corp., St. Louis, MO, USA), and D_2_O (>99.9%, Sigma-Aldrich Corp., St. Louis, MO, USA) were used as received. All other reagents and solvents were of analytical grade and were purified and/or dried by standard methods. Ultrapure water (Milli-Q, Millipore; Merck KGaA, Darmstadt, Germany) was used in all the experiments (synthesis and analysis).

### 3.2. Characterization of Wet Pomace 

The moisture content of WP was determined by drying at 105 °C for 48 h, until a constant weight was reached. The dried solid was named pomace-1 (P-1). Total dissolved solids (TDS) and total suspended solids (TSS) were determined from P-1 by stirring a suspension of 2 g in 100 mL of water for 2 h at rt followed by filtration through a 1.2 μm membrane. The amount of TDS was obtained after evaporation of the filtrate to dryness and drying at 105 °C, while the TSS was quantified after drying the filtration’s residue at 105 °C. The pH and the conductivity were determined on the TDS sample, and the ashes were quantified by heating P-1 in a furnace at 600 °C for 4 h [60]. The lipid content on P-1 was quantified after solid-liquid extraction with *n*-hexane in a Soxhlet apparatus during 4 h, following a standard procedure [61]. The dried residue was denoted as pomace-2 (P-2). The protein content was determined on P-2, following reported methods [62,63]. Soxhlet extraction of P-2 with EtOH/H_2_O (80:20 *v*/*v*) for 4 h yielded an alcoholic/aqueous extract containing low molecular weight sugars and phenols [64], and an extractive-free residue termed pomace-3 (P-3). Soluble phenols in the alcoholic/aqueous extract, expressed in gallic acid equivalents, were determined by an adapted Folin-Ciocalteau method [65], whereas the soluble sugars were quantified by the phenol/sulfuric acid method [64]. Lignin and holocellulose were determined on P-3 following the Klason method [66] and the chlorite method [67,68], respectively.

### 3.3. Typical Procedure for the Synthesis of WP-CDs

Pomace (P-2; 8.0 g) dispersed in water (50 mL) and ethylenediamine (EDA; 712 μL; EDA/P-2 mass ratio = 0.08) was charged into an inox vessel of a high-pressure reactor (Parr model 4560, Parr Instrument Company, Moline, IL, USA) equipped with pressure, temperature and stirring controllers (Parr model 4843, Parr Instrument Company, Moline, Ill, USA). The sealed contents were stirred (250 rpm), flushed through with nitrogen three times, left with an N_2_ pressure of 2 bar, and heated with a ramp rate of 15 °C/min until the desired temperature (200–300 °C) was reached. After achieving the set temperature, steady autogenous pressures of 15, 40, and 85 bar were developed, at 200 °C, 250 °C and 300 °C, respectively. After a certain dwell time (4–72 h), the reaction mixture was naturally cooled to room temperature (rt) and filtered through a 0.20 μm cellulose membrane under vacuum. The amount of carbon nanomaterials in the filtrate (designated as-synthesized WP-CDs) was gravimetrically determined after taking an aliquot of the filtrate to dryness and drying at 105 °C. For most of the experiments here reported, the above filtrate was further extracted with CH_2_Cl_2_ until the organic phase was colorless (~5 × 25 mL). The residual organic solvent in the aqueous phase was smoothly removed on a rotary evaporator, resulting in a brown aqueous solution of CDs (named as-purified WP-CDs). The filtration cake (wet hydrochar) was dried at 105 °C and quantified. Mass yields of CDs and hydrochar (HChar) were calculated as: mass yield (wt %) = m_CDs_ or m_HChar_/(m_P_ + m_EDA_) × 100. 

### 3.4. Cell Culture and Cell Viability 

L929 (mouse fibroblast) and HeLa (human cervical cancer) cell lines were cultured in Dulbecco’s Modified Eagle’s Medium, DMEM (GIBCO™, Thermo Fisher Scientific, Waltham, MA, USA), supplemented with 10% fetal bovine serum (FBS, GIBCO™, Thermo Fisher Scientific, Waltham, MA, USA) and 1% penicillin/streptomycin (GIBCO™, Thermo Fisher Scientific, Waltham, MA, USA) and maintained in a humidified atmosphere with 5% CO_2_ at 37 °C. All cell culture lines were maintained with routine subcultures (using TrypLE Express without phenol red, GIBCO™, Thermo Fisher Scientific, Waltham, MA, USA, for chemical detaching). Mammalian cell lines were counted with a Neubauer chamber.

The cytotoxicity effect of WP-CDs was evaluated by a resazurin-based assay using PrestoBlue™ reagent (Invitrogen, Carlsbad, CA, USA). Briefly, L929 and HeLa cell lines (purchased at European Collection of Authenticated Cell Cultures, Porton Down, Wiltshire, UK) were seeded in 96-well tissue cultured plates (Orange) at an initial density of 1 × 10^4^ cells per well and left overnight in a CO_2_ incubator (5%) at 37 °C. The cell medium (DMEM supplemented with 10% FBS and 1% penicillin-streptomycin) was then discarded and replaced with solutions of different WP-CDs amounts (0.5–1000 µg/mL) previously prepared in complete cell culture medium. Both cell lines were incubated for an additional 24 h period. Quantification of the cell viability was performed accordingly to the PrestoBlue^TM^ reagent kit protocol. The resazurin conversion into resorufin was monitored by measuring fluorescence intensity (excitation 530 nm, emission 590 nm) in a microplate reader (BMG Labtech, Polar Star Optima, Ortenberg, Germany) at 37 °C. Cell viability results (%) are expressed with reference to the controls. Viability assays were carried out in triplicate. An experiment without cells was also performed to assess the direct influence of WP-CDs on the cell viability results, using an aqueous solution of resazurin (10 vol%) and WP-CDs in the same concentration range (0.5–1000 µg/mL). Emission intensity was measured at 590 nm upon a 530 nm excitation after 24 h of incubation (duplicate assays).

### 3.5. Radical Scavenging Activity [55]

A series of solutions of WP-CD-5 were prepared at various concentrations (7.4–138 μg/mL) in absolute ethanol. To 1 mL solution of each CD concentration was added 2 mL of a freshly prepared ethanolic solution of DPPH (0.127 mM). The mixture was gently shaken and kept in the dark for 1 h at 25 °C. The absorption spectrum of each mixture was acquired, and the absorbance was read at 520 nm. The control samples were made by adding 1 mL EtOH to the DPPH solution. Assays were conducted in triplicate. The radical scavenging activity of WP-CDs towards DPPH was calculated as: DPPH scavenging activity/% = (A_0_ − A)/A_0_ × 100, where A_0_ is the absorbance of DPPH solution in the absence of CDs and A the absorbance of the mixture. The same procedure was used for determinations with ascorbic acid (concentration range: 0.67–16 μg/mL) and the alcoholic/aqueous extract of pomace-2 (concentration range: 20–100 μg/mL).

### 3.6. Instruments and Methods

FTIR spectra were acquired on a Bruker Vertex 70 (Bruker Optik GmbH, Ettlingen, Germany) in transmission mode with a spectral resolution of 2 cm^−1^, using OPUS ver. 5.5. software for data acquisition and processing. Pellets were prepared by grinding the WP-CDs, previously dried in a vacuum for at least 12 h at 105 °C with KBr. Tentative band assignments are indicated by the nature of the vibration: stretching (str), bending (ben), symmetric (sym) and asymmetric (asym), and their relative intensities (very weak (vw), w (weak), medium (m), strong (s) and very strong (vs)).

Micro-Raman measurements were carried out in a back-scattering micro-configuration, with a Renishaw InVia confocal Raman microscope (Renishaw plc, Gloucestershire, UK), using a 532 nm excitation and 100×, 50×, 20×, 10× and 5× objectives. The spectral resolution of the Raman spectrometer was ~ 1 cm^−1^. All Raman spectra were recorded at least three times for each WP-CDs sample in different locations, being all spectra presented truly representative. Data acquisition and processing was performed with the Renishaw software Wire 4. Baseline correction was done where needed with Wire 4 software or with LabSpec software from Jobin Yvon. The multiple peak fit of Raman data was carried out with Origin 9.0 software.

XPS analysis was performed with a non-monochromatic dual anode spectrometer XSAM800 from KRATOS (KRATOS, Manchester, UK). Mg Kα X-rays were used; spectra were acquired in FAT mode with a power of 120 W and a pass energy of 20 eV and TOA = 45°. An aqueous WP-CDs dispersion was used to coat a Si substrate (with no particular cleaning procedure) by successive drop/solvent evaporation at 0.2 bar at rt. The substrate was completely coated since no silicon was detected. No flood gun was used for charge compensation. The charge shift was corrected using the binding energy (BE) of aromatic C-C or C-H, centered at 284.7 eV, as reference. Source satellites were subtracted. Pseudo-Voight profiles were used for peak fitting with the software XPSPeak4.1. Shirley baselines were used. The sensitivity factors used for the quantification analysis were those of the library of Vision 2 for Windows, Version 2.2.9 from KRATOS.

^1^H NMR and ^13^C NMR spectra were collected on a Brüker AVANCE II+ spectrometer (Bruker BioSpin AG, Fällanden, Switzerland), working at 400.130 MHz and 100.577 MHz, respectively. The reported chemical shifts (δ/ppm) are internally referenced to residual non-deuterated solvent (^1^H NMR, D_2_O, 4.79 ppm) and DMF as a residual solvent in D_2_O (^13^C NMR, 165.53 ppm). All NMR spectra were recorded at rt (~25 °C). Phase corrections and editing were done with MestReNova software (ver. 14.1.2-25024) from Mestrelab Research S. L., 2020.

The transmission electron microscopy (TEM) was carried out in a Hitachi H-8100 microscope (Hitachi High-Tech Corporation, Tokyo, Japan) operating at 200 kV (lantanium hexaboride filament), with a point-to-point resolution of 2.7 Å using a 200-mesh copper grid covered with a Formvar Carbon film, at MicroLab, Universidade de Lisboa, Portugal. The scanning transmission electron microscopy (STEM) was performed in a JEOL JEM 2010F microscope (JEOL Ltd., Tokyo, Japan) operating at 200 kV, at C.A.C.T.I., Universidad de Vigo, Spain, using high-angle annular dark field (HAADF) and bright field (BF) detectors.

Aqueous solutions of WP-CDs were deposited in the support and left to evaporate naturally at ca. 20 °C. The diameter of the particles was estimated by ImageJ software [69].

The elemental analysis (CHNS) was performed by the combustion method (combustion performed at 1015 °C during 900 s with an oxygen flux of 15 mL/min) on a Carlo Erba EA 1108 analyzer (Carlo Erba, Milan, Italy) at C.A.C.T.I., Universidad de Vigo, Spain. 

Ground-state UV-Vis spectra were recorded on a Jasco UV V-750 spectrophotometer (Jasco Inc., Tokyo, Japan) using 1-cm quartz cells at 25 °C. Jasco Spectra Manager II software (ver. 2.08.01) was used for data acquisition and analysis. Steady-state fluorescence spectra were acquired on a Perkin Elmer LS45 fluorimeter (PerkinElmer Life and Analytical Sciences, Shelton, USA) using a 1-cm quartz cuvette in a right angle (RA) setup at 25 °C, in air-equilibrated conditions. Data acquisition and processing was done with BioLight Luminescence Systems software ver. 1.01.02.

Fluorescence quantum yields (QY) of aqueous solutions of WP-CDs at 25 °C were obtained by the slope method [70], using quinine sulphate in 0.01 M H_2_SO_4_ (QY = 0.54; air equilibrated conditions, RA geometry) as the standard. The absorbances of the samples and reference were kept below 0.05 at the excitation wavelength to prevent homo-inner filter effects. The excitation and emission spectra of all samples were recorded under the same operating settings. Fluorimetric experiments were carried out in water, using WP-CDs at a concentration of 0.1 mg/mL, except if noticed otherwise. For photostability tests, a phosphate buffer solution (50 mM, pH = 7.2) was used. In all cases, the data was acquired in RA geometry.

Time-resolved fluorescence was measured by the single-photon timing (SPT) method using a mode locked DPSS Nd:YVO_4_ green laser (Vanguard 2000-HM532, Spectra Physics Inc., Santa Clara, CA, USA) synchronously pumping a cavity dumped dye laser (701, Coherent, delivering frequency-doubled 3–4 ps pulses of about 40 nJ/pulse at 3.4 MHz) working with 4-(dicyanomethylene)-2-methyl-6-(4-dimethylaminostyryl)-4H-pyran (DCM). Emission light was detected by a Hamamatsu 2809U-01 microchannel plate photomultiplier (Hamamatsu Photonics K.K., Ichino-cho, Higashi-ku, Japan) at 430 nm, using an excitation wavelength of 340 nm. For two-photon absorption (TPA) measurements, an excitation wavelength of 620 nm was used. Experimental intensity decays were fitted to the multi-exponential model:

(1) $$I\left(t\right)=\underset{i}{\sum }\alpha_{i}\exp\left(-t/\tau_{i}\right)$$

where $\alpha_{i}$ are the amplitudes of the component decays at *t* = 0. A DeltaFlex lifetime system (Horiba Scientific, Northampton, UK) was also used under 405 nm excitation wavelength, using a BDS-405-SM-FBE ps diode laser (Becker & Hickl, GmbH, Germany).

The pHs of aqueous/buffered solutions were determined at ca. 25 °C with a pH VWR pHenomenal^®^ UM 6100L equipped with a pH electrode phenomenal 221 (VWR International BVBA, Leuven, Belgium).

## 4. Conclusions

Biocompatible carbon-based nanomaterials were synthesized from wet pomace, a highly abundant biomass waste from industrial olive oil production. WP-CDs are photostable blue emitters with high fluorescence QYs, armed with up-conversion photoluminescence capabilities, making them suitable candidates for applications in bioimaging, (bio)sensing, and photodynamic therapies, among other uses. Useful correlations between the CDs structures and their optical properties have been established through spectroscopic techniques. A few noteworthy remarks are in order: (i) the fluorescence of WP-CDs is a collective property of constituent nanoparticles, each comprising its own emitter of varying size and structure; (ii) the emitters are composed by fused arene/heteroarene moieties and aliphatic regions, linked mainly to carboxylate groups, which render the whole particle hydrophilic and soluble in polar solvents; (iii) the different sizes of the emitters, each with its own conjugation length and attached functionalities, may be at the origin of the observed excitation wavelength-dependent emissions; and (iv) inter-particle aggregation causes a huge reduction on the fluorescence QY, accompanied by a strong redshift of emission.

Work is ongoing to establish the actual sizes of emissive domains and their relation to the particle’s dimension, through size-fractioning and fluorescence anisotropy measurements, to further enlighten their nature and harness the full potential of these materials.

## Figures and Tables

**Table 1:** Physico-chemical properties of wet pomace.

Properties	Weight %
Moisture ^1^	68.4 ± 3.1
TSS ^2,4^	82.9 ± 0.2
TDS ^2,4^	17.1 ± 0.2
Ash ^2,4^	4.8 ± 0.3
pH ^1,3^	5.5 ± 0.2
Conductivity (μS/cm) ^1,3^	567.3 ± 1.2

^1^ Triplicate assays. ^2^ Duplicate assays. ^3^ Determined on TDS at 25 °C. ^4^ Determined on a dry basis. TSS: Total suspended solids. TDS: Total dissolved solids.

**Table 2:** Macro-organic constituents of wet pomace.

Constituent	Weight % (Dry Basis)
Pomace oil extract ^1,4^	12.7 ± 0.6
Alcoholic extract ^2,5^	15.8 ± 0.02
Soluble phenols ^4^	2.3 ± 0.3
Soluble sugars ^4^	3.3 ± 0.1
Insoluble lignin ^3,5^	33.4 ± 0.9
Soluble lignin ^3,5^	2.5 ± 0.5
Holocellulose ^3,5^	40.0 ± 0.3
Protein ^2,5^	5.4 ± 0.6

^1^ Determined in P-1. ^2^ Determined in P-2. ^3^ Determined in P-3. ^4^ Triplicate (or higher) assays. ^5^ Duplicate assays.

**Table 3:** Mass yields of as-synthesized CDs and hydrochars (HChar), and the corresponding CDs’ quantum yields ^1^.

Pomace	Temp. (°C)	CDs wt %	HChar wt %	QY ^2^
P-1	250	18.8	25.5	0.14
P-2	200	22.9	48.5	0.095
	250	19.6	25.8	0.145
	300	13.0	17.5	0.064
P-3	200	17.4	51.0	0.096
	250	18.3	26.8	0.15
	300	14.7	25.8	0.14
P-Ind ^3^	250	22.4	19.4	0.16

^1^ Typical reaction conditions: [P] = 0.04 g/mL; ethylenediamine (EDA)/P mass ratio = 0.08; 4 h. ^2^ QY determined under a 380 nm excitation wavelength. ^3^ [P-Ind] = 0.16 g/mL. CDs: carbon dots. HChar: hydrochar. QY: quantum yield.

**Table 4:** Characterization of decomposed Raman modes for WP-CDs-1 and 5.

WP-CDs-1					WP-CDs-5			
Mode	Shift (cm^−1^) ^1^	FWHW (cm^−1^)	Area (%)	I_D_/I_G_^2^	Shift (cm^−1^) ^1^	FWHW (cm^−1^)	Area (%)	I_D_/I_G_^2^
C-H rings	1177	76	5.0	0.75	1182	99	5.0	1.19
D	1309	143	28.2		1315	155	37.2	
Kekulé	1447	176	33.1		1472	178	35.8	
G	1569	113	29.8		1570	99	19.9	
C=O	1690	67	3.8		1679	75	2.0	

^1^ Band peak maximum after Gaussian fit. ^2^ Estimated after Gaussian fitting using peak heights. FWHW: Full width at half maximum. I_D_/I_G_: ratio of intensities of D (I_D_) and G (I_G_) bands.

## Data Availability

Not applicable.

## Supplementary Materials

The following supporting information can be downloaded at: https://www.mdpi.com/article/10.3390/molecules27196768/s1; Figure S1: UV-Vis and fluorescence spectra of WP-CDs-P1 to P3 and P-Ind; Figures S2–S5 and Tables S1–S3: FTIR analysis; Figure S6 and Table S4: Raman analysis; Tables S5 and S6 and Figure S7: XPS analysis; Figure S8: UV-Vis analysis; Tables S7 and S8: Quantum yields and lifetimes; Figure S9: Emission dependence on excitation wavelength; Figure S10: Quenching of emission by an external quencher; Figure S11: Photostability; Figure S12: Emission vs. pH; Table S9: Lifetime vs. concentration; Figure S13: Effect of WP-CDs on resazurin reduction.

## Sample Availability

Samples of some reported nanomaterials are available from the authors upon request.

## Abbreviations

ACQ	Aggregation-caused quenching
BF	Bright field [detector]
CDs	Carbon dots
DPPH	2,2-Diphenyl-1-picrylhydrazyl [radical]
EDA	Ethylenediamine
FTIR	Fourier transform infrared [spectroscopy]
FWHM	Full width at half maximum
HAADF	High-angle annular dark field [detector]
HChar	Hydrochar
HTC	Hydrothermal carbonization
NMR	Nuclear magnetic resonance [spectroscopy]
P	Pomace
PL	Photoluminescence
QY	Quantum yield
SC-QDs	Semi-conductor quantum dots
SEC	Size-exclusion chromatography
SPT	Single-photon timing [method]
STEM	Scanning transmission electron microscopy
TDS	Total dissolved solids
TEM	Transmission electron microscopy
TPA	Two-photon absorption
TSS	Total suspended solids
WP	Wet pomace
XPS	X-ray photoelectron spectroscopy
