# Supplementary material for: Luminescent Carbon Dots from Wet Olive Pomace: Structural Insights, Photophysical Properties and Cytotoxicity
Source: Molecules. 2022 Oct 10;27(19):6768. doi: 10.3390/molecules27196768 (PMC9573145; doi:10.3390/molecules27196768)
Supplement: Supplementary file 1 [file molecules-27-06768-s001.zip › molecules-1930096-supplementary.pdf]

Luminescent Carbon Dots from Wet Olive Pomace: structural insights, photophysical properties and cytotoxicity

Diogo A. Sousa [1,2,3], Luís F. V. Ferreira, [3] Alexander A. Fedorov, [3] Ana M. B. do Rego, [3] Ana M. Ferraria, [3] Adriana B. Cruz, [3] Mário N. Berberan-Santos, [3] and José V. Prata [1,2,*]

[1] Department of Chemical Engineering, Instituto Superior de Engenharia de Lisboa, Instituto Politécnico de Lisboa, 1959-007 Lisbon, Portugal.
[2] CQ-VR-Centro de Química-Vila Real, Universidade de Trás-os-Montes e Alto Douro, 5001-801 Vila Real, Portugal.
[3] BSIRG-iBB-Institute for Bioengineering and Biosciences, and Associate Laboratory i4HB—Institute for Health and Bioeconomy, Instituto Superior Técnico, Universidade de Lisboa, 1049-001 Lisbon, Portugal.
* Correspondence: jvprata@deq.isel.ipl.pt

Supplementary Materials

Table of Contents

UV-Vis and fluorescence spectra of WP-CDs-P1 to P3 and P-Ind: Figure S1

FTIR analysis: Figures S2-S5 and Tables S1-S3

Raman analysis: Figure S6 and Table S4

XPS analysis: Tables S5-S6 and Figure S7

UV-Vis analysis: Figure S8

Quantum yields and lifetimes: Tables S7-S8

Emission dependence on excitation wavelength: Figure S9

Quenching of emission by an external quencher: Figure S10

Photostability: Figure S11

Emission vs pH: Figure S12

Lifetime vs concentration: Table S9

Effect of WP-CDs on resazurin reduction: Figure S13

UV-Vis and fluorescence emission spectra of WP-CDs-P1 to P-3 and P-Ind

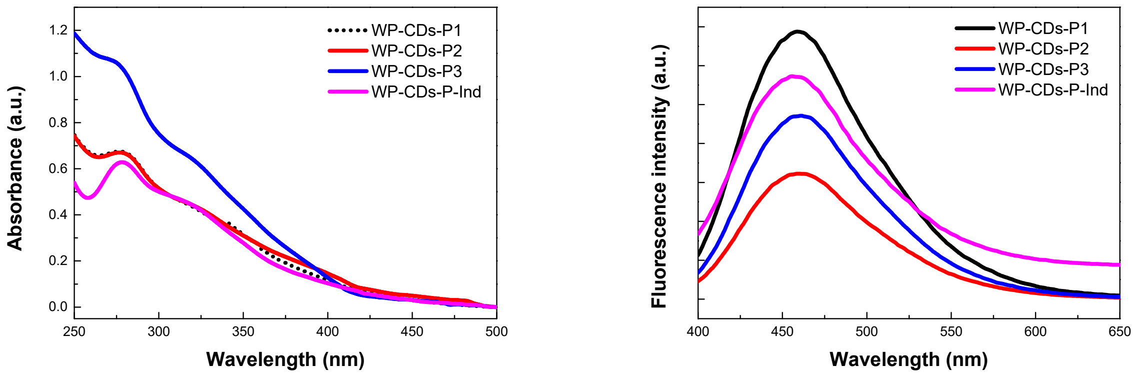

(**a**) (**b**)

Figure S1. UV–Vis (**a**) and emission (**b**) spectra of aqueous solutions of WP-CDs prepared at 250 °C using a [P] = 0.04 g/mL, an EDA/P mass ratio = 0.08, and a 4 h heating period; for P-Ind, a concentration of 0.16 g/mL was used. Excitation at 380 nm. Emission spectra are offset for clarity's sake.

FTIR analysis

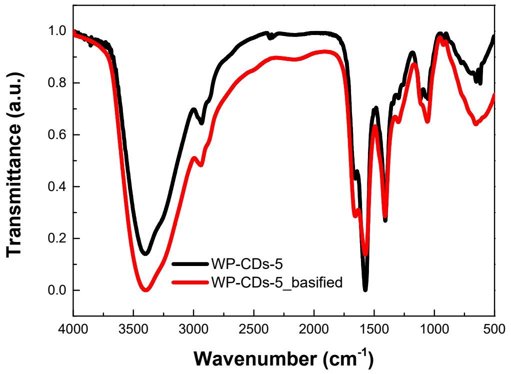

Figure S2. FTIR spectrum of pristine WP-CDs-5 and that of the same sample after it has been acidified, boiled, and basified to pH = 8.7 (WP-CDs-5_basified).

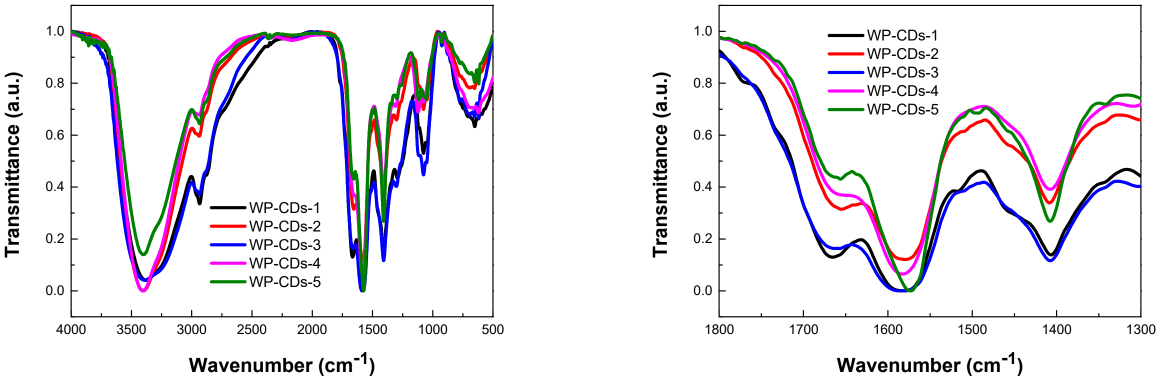

(**a**) (**b**)

Figure S3. (**a**) Overlayed FTIR spectra of WP-CDs synthesized under several heating periods (4 h, 8 h, 16 h, 32 h, and 72 h), using a [P-2] = 0.16 g/mL, an EDA/P-2 mass ratio = 0.08, at 250 °C. (**b**) The same spectra amplified in the region between 1800-1300 cm^{-1}.

FTIR analysis (cont.)

Table S1. FTIR data for WP-CDs as a function of dwell time.[1]

CDs	Dwell Time (h)	υ_1 (cm^{-1})	υ_2 (cm^{-1})	υ_3 (cm^{-1})	$I\upsilon_2/I\upsilon_1$ [2]	QY [3]
WP-CDs-1	4	1665	1583	1407	1.15	0.15
WP-CDs-2	8	1658	1582	1408	1.29	0.17
WP-CDs-3	16	1662	1582	1408	1.20	0.19
WP-CDs-4	32	1660	1582	1408	1.49	0.22
WP-CDs-5	72	1660	1572	1408	1.78	0.23

[1] Typical reaction conditions: [P-2] = 0.16 g/mL; EDA/P-2 mass ratio = 0.08, at 250 ºC. [2] Ratio of normalized band intensities. [3] QY determined with excitation at 340 nm.

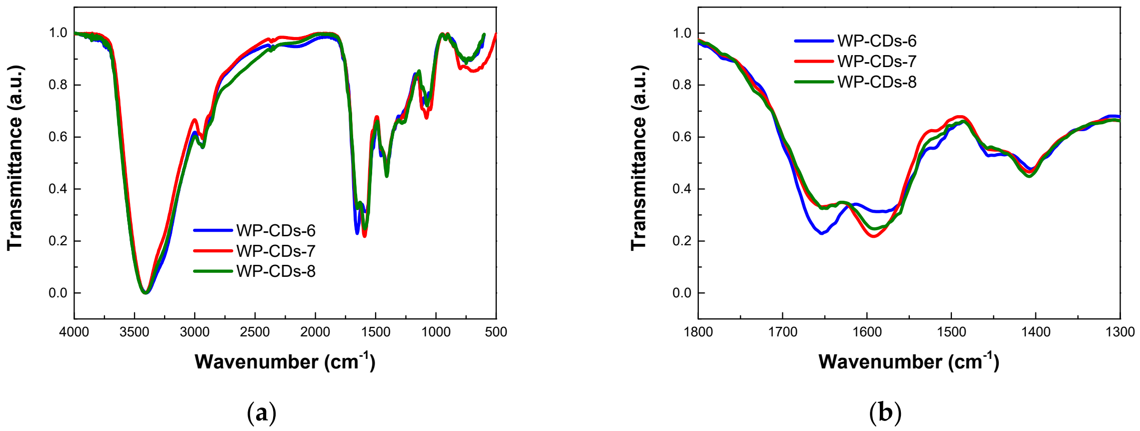

Figure S4. (**a**) FTIR spectra of WP-CDs synthesized at 200 ºC, 250 ºC and 300 ºC during 4 h, using a [P-2] = 0.04 g/mL, and an EDA/P-2 mass ratio = 0.08. (**b**) The same spectra amplified in the region between 1800-1300 cm^{-1}.

Table S2. FTIR data for as-synthesized WP-CDs as a function of temperature.[1]

CDs	Temp.(ºC)	υ_1 (cm^{-1})	υ_2 (cm^{-1})	υ_3 (cm^{-1})	$I\upsilon_2/I\upsilon_1$ [2]	QY [3]
WP-CDs-6	200	1654	1582	1405	0.89	0.095
WP-CDs-7	250	1655	1593	1406	1.17	0.145
WP-CDs-8	300	1652	1592	1408	1.12	0.064

[1] Typical reaction conditions: [P-2] = 0.04 g/mL; EDA/P-2 mass ratio = 0.08; 4 h. [2] Ratio of normalized band intensities. [3] QY determined with excitation at 380 nm.

FTIR analysis (cont.)

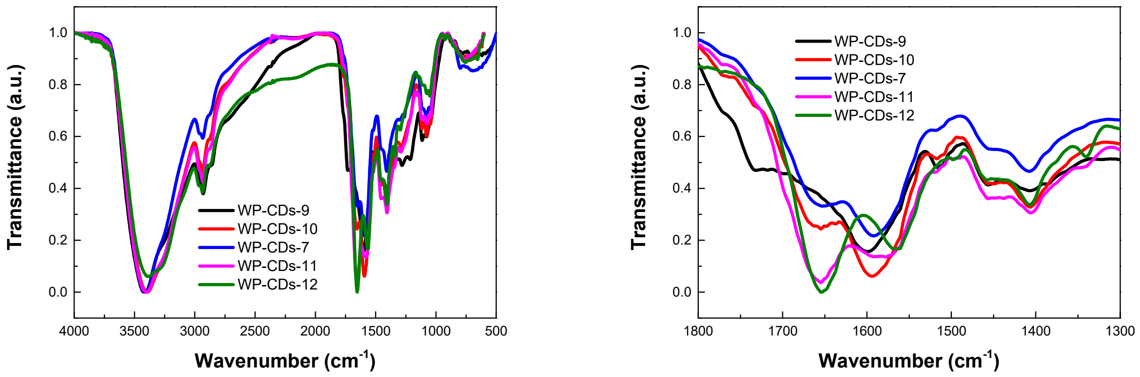

(**a**) (**b**)

Figure S5. (**a**) FTIR spectra of WP-CDs synthesized with different EDA/P-2 mass ratios (0, 0.04, 0.08, 0.16, and 0.32) at 250 °C during 4 h, using a [P-2] = 0.04 g/mL. (**b**) The same spectra amplified in the region between 1800-1300 cm^{-1}.

Table S3. FTIR data for as-synthesized WP-CDs as a function of EDA/P mass ratio. [1]

CDs	EDA/P mass ratio	υ_1 (cm^{-1})	υ_2 (cm^{-1})	υ_3 (cm^{-1})	$I\upsilon_2/I\upsilon_1$ [2]	QY [3]
WP-CDs-9	0	1663	1599	1408	1.35	0.055
WP-CDs-10	0.04	1655	1594	1406	1.25	0.13
WP-CDs-7	0.08	1655	1593	1406	1.17	0.145
WP-CDs-11	0.16	1655	1586	1405	0.90	0.12
WP-CDs-12	0.30	1654	1566	1407	0.84	0.10

[1] Typical reaction conditions: [P-2] = 0.04 g/mL; 250 °C; 4 h. [2] Ratio of normalized band intensities.
[3] QY determined with excitation at 380 nm.

Raman analysis

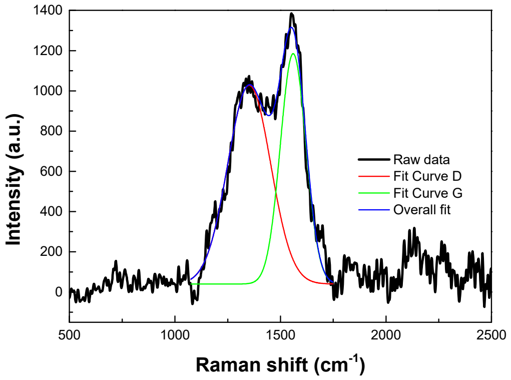

(**a**)

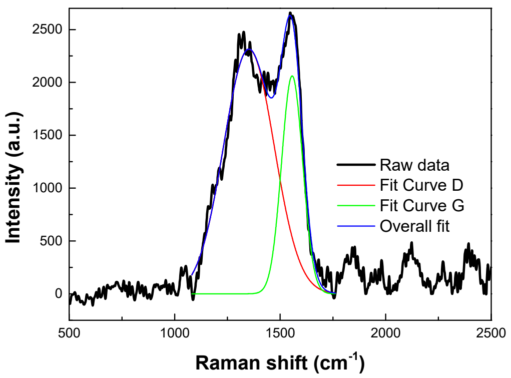

(**b**)

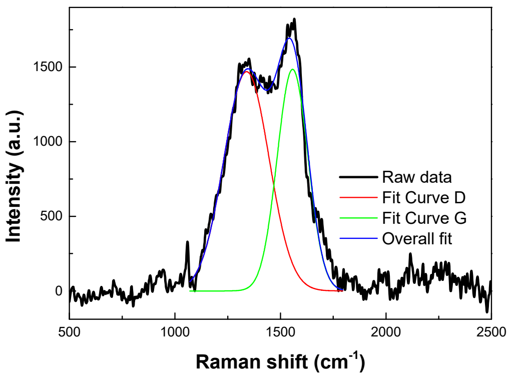

(**c**)

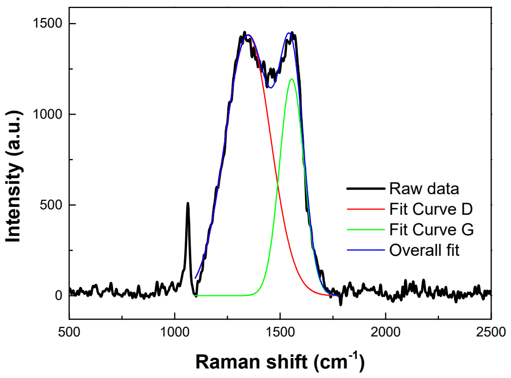

(**d**)

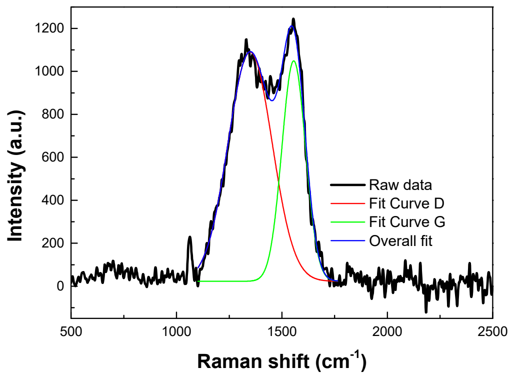

(**e**)

Figure S6. Raman spectra of WP-CDs prepared under (**a**) 4 h (WP-CDs-1) (**b**) 8 h (WP-CDs-2), (**c**) 16 h (WP-CDs-3), (**d**) 32 h (WP-CDs-4), and (**e**) 72 h (WP-CDs-5) of heating, using a [P-2] = 0.16 g/mL, an EDA/P-2 mass ratio = 0.08, at 250 °C. Curves corresponding to D and G bands obtained by Gaussian fitting.

Raman analysis (cont.)

Table S4. D and G bands for WP-CDs as a function of dwell time, FWHW, and I_D/I_G ratios.

**CDs**	**Dwell Time (h)** [1]	**D band (cm^{-1})** [2]	**D band FWHW (cm^{-1})**	**G band (cm^{-1})** [2]	**G band FWHW (cm^{-1})**	**I_D/I_G** [3]
WP-CDs-1	4	1352	239	1560	143	0.86
WP-CDs-2	8	1352	282	1557	118	1.12
WP-CDs-3	16	1339	251	1557	172	0.99
WP-CDs-4	32	1348	254	1555	137	1.20
WP-CDs-5	72	1349	245	1556	132	1.04

[1] Typical reaction conditions: [P-2] = 0.16g/mL; EDA/P-2 mass ratio = 0.08; 250 °C. [2] Band peak maximum after a Gaussian fit. [3] Estimated after Gaussian fitting using peak heights.

XPS analysis

Table S5. Corrected binding energies (BE) and atomic concentrations [At. conc. %] for all detected elements in various spectral regions, and the corresponding assignments.

XPS regions	BE ± 0.1 eV [At. conc. %]		Assignments [1]
	WP-CDs-3	WP-CDs-5	
C 1s	284.7 [40.0]	284.7 [33.2]	***C***-***C*** and ***C***-H sp^2 (sp^3 included, at 285 eV)
	286.0 [19.7]	285.9 [15.6]	***C***-N (286.0); ***C***-O (286.6)
	287.8 [4.8]	287.8 [9.2]	***C***=O and C_{aryl}–***C***(=O)O^- [See text]
K $2p_{3/2}$	292.5 [1.8]	292.5 [2.4]	K^+
K $2p_{1/2}$	295.3 [0.9]	295.2 [1.2]	
O 1s	531.0 [10.0]	530.8 [17.4]	R-C=***O*** (R = C-aryl or N) and C_{aryl}-C(=***O***)O^-
	532.4 [14.4]	532.3 [11.7]	***O***-C and C-***O***-C
N 1s	399.1 [2.0]		pyridinic *N*
	400.0 [3.2]	399.5 [7.4]	pyrrolic, aryl-*N*H and *N*-C=O (probably mixed with pyridinic peak in WP-CDs-5)
	401.4 [1.4]		Protonated or H-bonded amines
Cl $2p_{3/2}$	197.7 [1.3]	197.6 [0.16]	Cl^-
Cl $2p_{1/2}$	199.3 [0.6]	199.2 [0.08]	
Cl $2p_{3/2}$		200.6 [0.03]	Cl-C
Cl $2p_{1/2}$		202.2 [0.01]	
Na 1s		1070.5 [1.6]	Na^+

[1] Spectral assignments based on references [1-3].

Table S6. Overall XPS atomic concentrations (%) and corresponding weight % (computed from XPS at. conc. %).

Element	At. conc. (%)		Wt (%)	
	WP-CDs-3	WP-CDs-5	WP-CDs-3	WP-CDs-5
**C**	64.4	58.0	54.1	47.9
**K**	2.7	3.6	7.3	9.8
**O**	24.4	29.1	27.4	32.0
**N**	6.6	7.4	6.4	7.1
**Cl**	1.9	0.3	4.7	0.7
**Na**	-	1.6	-	2.5

XPS analysis (cont.)

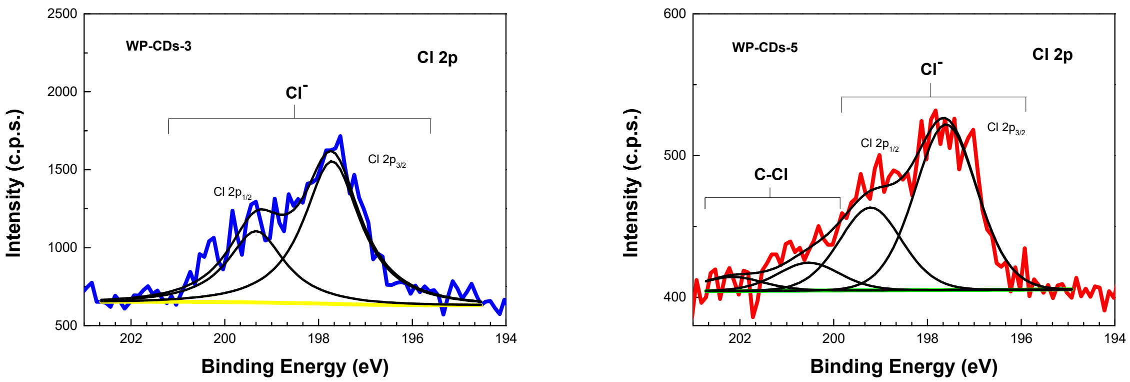

(**a**) (**b**)

Figure S7. XPS spectral regions of Cl 2p of (**a**) WP-CDs-3 and (**b**) WP-CDs-5.

UV-Vis analysis

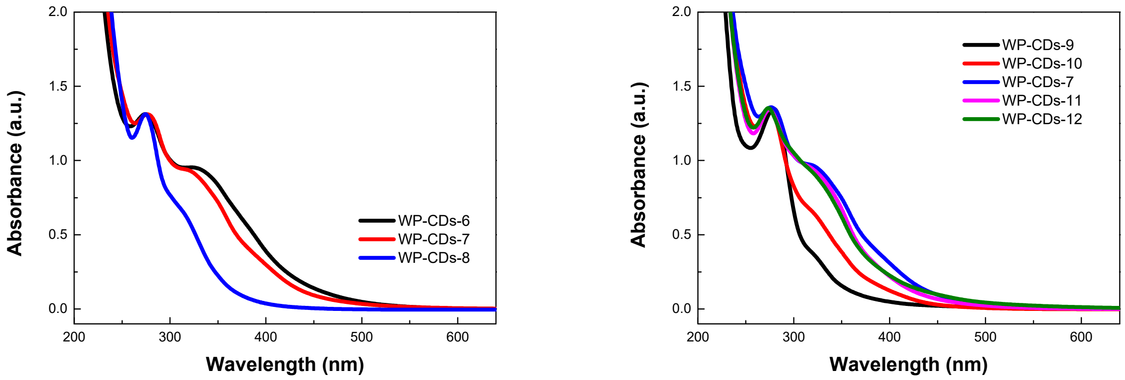

(**a**) (**b**)

Figure S8. UV–Vis spectra (normalised at 275 nm) of aqueous solutions of WP-CDs (0.1 mg/mL) prepared (**a**) at 200 °C (WP-CDs-6), 250 °C (WP-CDs-7), and 300 °C (WP-CDs-8), using a [P-2] = 0.04 g/mL, an EDA/P-2 mass ratio = 0.08, under a 4 h heating period; and (**b**) at various EDA/P-2 mass ratios (0 to 0.30), by heating for 4 h at 250 °C using a [P-2] = 0.04 g/mL.

Quantum yields and lifetimes

Table S7. Quantum yields of WP-CDs.

CDs	Unpurified samples [1]		Purified samples [2]	
	Excitation wavelength (nm)		Excitation wavelength (nm)	
	340	380	340	380
WP-CDs-1	0.13	0.19	0.15	0.19
WP-CDs-2	0.16	0.19	0.17	0.23
WP-CDs-3	0.165	0.17	0.19	0.20
WP-CDs-4	0.19	0.17	0.22	0.22
WP-CDs-5	0.23	0.21	0.23	0.165
WP-CDs-6	-	0.095	-	-
WP-CDs-7	-	0.145	-	-
WP-CDs-8	-	0.064	-	-
WP-CDs-9	-	0.055	-	-
WP-CDs-10	-	0.13	-	-
WP-CDs-11	-	0.12	-	-
WP-CDs-12	-	0.10	-	-

[1] Determined from aqueous solutions directly obtained from the reaction mixture after membrane (0.2 μm) filtration (as-synthesized WP-CDs). [2] Determined from the previous samples after solvent extraction (as-purified WP-CDs).

Table S8. Multi-exponential analysis of as-purified WP-CDs decays. [1]

CDs	f_1 [2]	τ_1	f_2	τ_2	f_3	τ_3	τ_{ave}	χ^2
WP-CDs-1	9.3	0.87	36.0	4.2	54.7	12.9	8.7	1.39
WP-CDs-2	7.8	1.07	40.5	4.6	51.7	13.1	8.7	1.10
WP-CDs-3	6.4	0.82	29.5	4.4	64.1	13.7	10.1	1.28
WP-CDs-4	6.5	1.13	36.9	4.7	56.7	13.4	9.4	1.08
WP-CDs-5	5.1	0.84	30.2	4.5	64.7	13.7	10.2	1.30
WP-CDs-7	7.0	0.61	33.6	3.5	59.4	11.9	8.3	1.38
WP-CDs-9	13.8	0.78	36.6	4.0	49.6	13.8	8.4	1.31

[1] Measurements obtained under 340 nm excitation and monitored at 430 nm, except for entries 2 and 4 which were observed at 460 nm. [2] Fractional contributions calculated from $f_i = \alpha_i \tau_i / \Sigma \alpha_i \tau_i$, where α_i are the pre-exponential factors (amplitudes of the component decays at $t = 0$).

Emission dependence on excitation wavelength

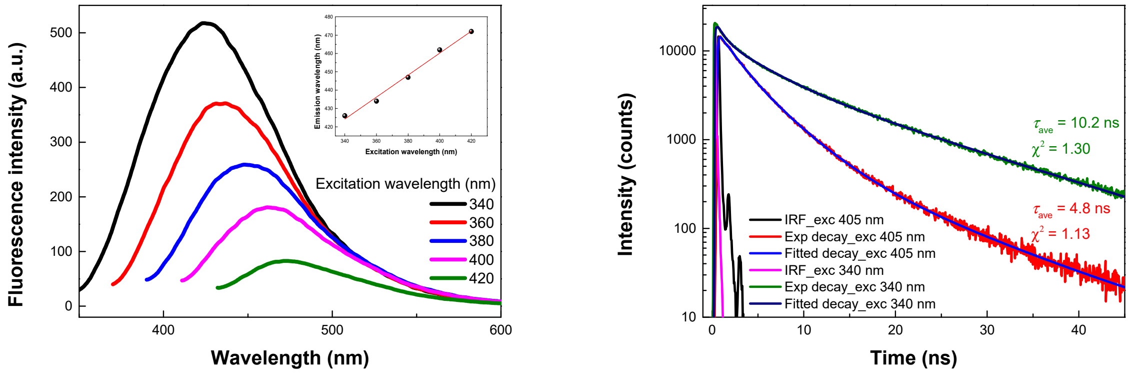

(**a**) (**b**)

Figure S9. (**a**) Fluorescence emission of aqueous solutions of WP-CDs-5 (0.1 mg/mL) upon excitation at wavelengths from 340 to 420 nm (Inset: linear dependence of emission on the excitation wavelength) and (**b**) Intensity decays of WP-CDs-5 excited at 340 nm and 405 nm.

Quenching of emission by an external quencher

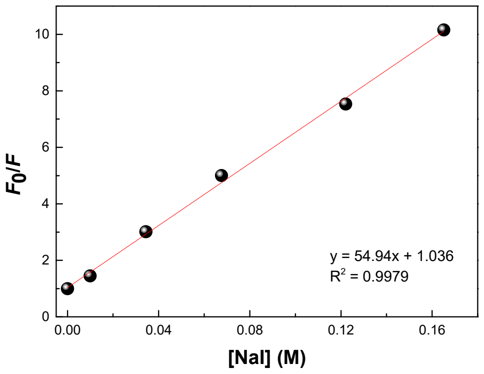

Figure S10. Stern-Volmer plot of the quenching of WP-CDs-5 (0.1 mg/mL) emission upon addition of NaI in sodium thiosulphate solution (1.0 x 10^{-4} M). Excitation at 340 nm.

Photostability

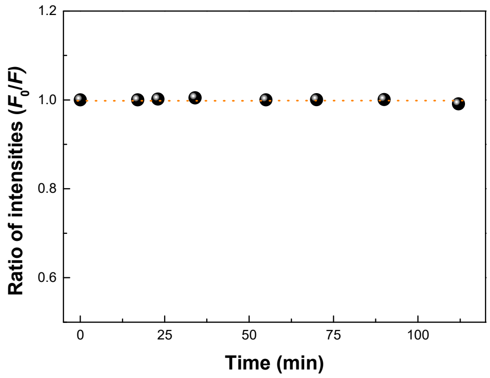

Figure S11. Plot of intensities' ratio of fluorescence emission spectra of buffered solutions of WP-CDs (0.1 mg/mL) upon continuous irradiation (up to 1 h 50 min) at a wavelength of 340 nm. Dotted line drawn as an eye guide.

Emission intensity vs pH

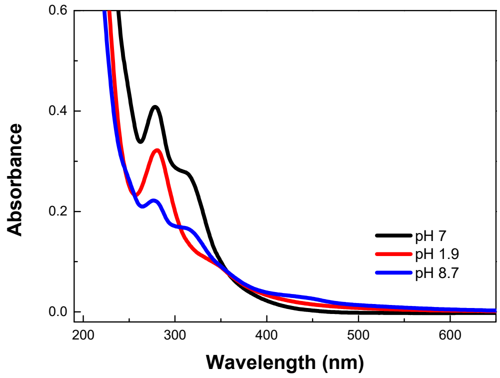

(**a**)

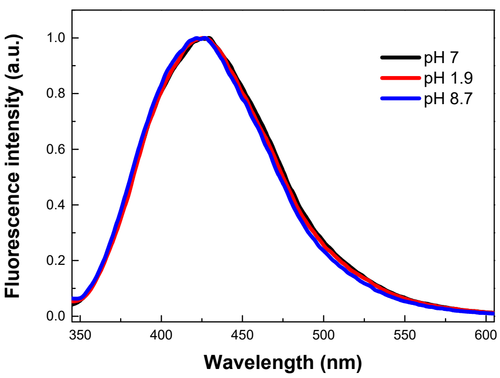

(**b**)

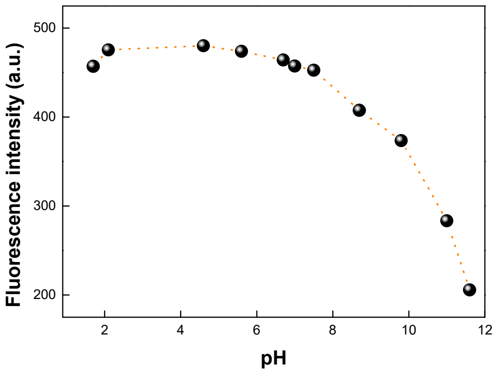

(**c**)

Figure S12. (**a**) UV-Vis spectra of aqueous solutions of WP-CDs-5 (0.1 mg/mL) at pH 1.9, 7 and 8.7 and (**b**) the corresponding normalised emission spectra (excitation at 340 nm); (**c**) variation of fluorescence intensity of the same WP-CDs-5 solutions on changing the pH from 1.7 to 11.6. Dotted line drawn as an eye guide.

Lifetime vs concentration

Table S9. Three-component exponential analysis of WP-CDs-5 decay as a function of concentration. [1]

[CDs] (mg/mL)	Observation wavelength (nm)	f_1 [2]	τ_1	f_2	τ_2	f_3	τ_3	τ_{ave}	χ^2
0.1	430	4.2	0.68	32.2	4.2	63.6	13.5	10.0	1.29
0.5	435	5.8	0.80	33.6	4.1	60.6	13.1	9.3	1.24
1.0	445	6.5	0.79	36.5	4.3	57.0	12.7	8.9	1.28
2.0	460	5.9	0.73	34.7	4.0	59.4	11.9	8.5	1.18
5.0	500	5.8	0.68	37.5	3.9	56.7	10.6	7.5	1.11

[1] Measurements obtained under 340 nm excitation. [2] Fractional contributions calculated from $f_i = \alpha_i \tau_i / \Sigma \alpha_i \tau_i$, where α_i are the pre-exponential factors (amplitudes of the component decays at $t = 0$).

Effect of WP-CDs on resazurin reduction

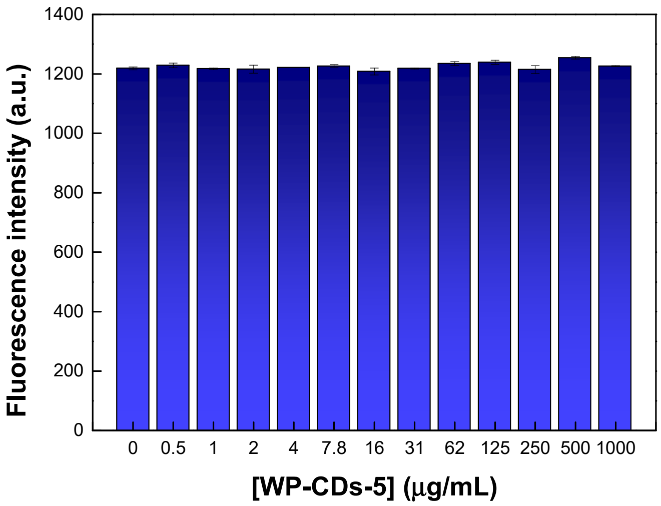

Figure S13. Fluorescence emission intensity of resazurin (10 vol %) monitored at 590 nm in the presence of increasing concentrations of WP-CDs-5 (0.5 – 1000 μg/mL), after 24 h of incubation. Excitation at 530 nm.

References

1. Library of Vision 2 for Windows, Version 2.2.9 from KRATOS; KRATOS: Manchester, UK, 2011.
2. Naumkin, A.V.; Kraut-Vass, A.; Gaarenstroom, S.W.; Powell, C.J. NIST X-ray Photoelectron Spectroscopy Database, NIST Standard Reference Database 20, Version 4.1.; National Institute of Standards and Technology: Gaithersburg, MD, USA, 2012.
3. Beamson, G.; Briggs, D. *High Resolution XPS of Organic Polymers*; The Scienta ESCA300 Database; John Wiley & Sons, Ltd: Chichester, UK, 1992.
